# Investigating Solar Wind Outflows from Open–Closed Magnetic Field Structures Using Coordinated Solar Orbiter and Hinode Observations

**DOI:** 10.1007/s11207-025-02438-8

**Published:** 2025-04-03

**Authors:** Nawin Ngampoopun, Roberto Susino, David H. Brooks, Roberto Lionello, Lucia Abbo, Daniele Spadaro, Deborah Baker, Lucie M. Green, David M. Long, Stephanie L. Yardley, Alexander W. James, Marco Romoli, Silvio M. Giordano, Aleksandr Burtovoi, Federico Landini, Giuliana Russano

**Affiliations:** 1 Mullard Space Science Laboratory, University College London, Holmbury St. Mary, Dorking, Surrey, RH5 6NT UK; 2 National Institute for Astrophysics, Astrophysical Observatory of Torino, Via Osservatorio 20, I-10025 Pino Torinese, Italy; 3 Computational Physics Inc., Springfield, VA 22151 USA; 4 Predictive Science Inc., San Diego, CA 92121 USA; 5 National Institute for Astrophysics, Astrophysical Observatory of Catania, Via Santa Sofia 78, I-95123 Catania, Italy; 6 Centre for Astrophysics & Relativity, School of Physical Sciences, Dublin City University, Glasnevin Campus, Dublin, D09 V209 Ireland; 7 Astronomy & Astrophysics Section, Dublin Institute for Advanced Studies, Dublin, D02 XF86, Ireland; 8 Department of Mathematics, Physics and Electrical Engineering, Northumbria University, Ellison Place, Newcastle Upon Tyne, NE1 8ST UK; 9 Donostia International Physics Center (DIPC), Paseo Manuel de Lardizabal 4, 20018 San Sebastián, Spain; 10 Department of Physics and Astronomy, University of Florence, Via Giovanni Sansone 1, I-50019 Sesto Fiorentino, Italy; 11 National Institute for Astrophysics, Astronomical Observatory of Capodimonte, Salita Moiariello 16, I-80131 Napoli, Italy

## Abstract

ESA/NASA’s Solar Orbiter (SO) enables us to study the solar corona at closer distances and from different perspectives, which helps us to gain significant insights into the origin of the solar wind. In this work, we present the analysis of solar wind outflows from two locations: a narrow open-field corridor and a small, mid-latitude coronal hole. These outflows were observed off-limb by the Metis coronagraph onboard SO and on-disk by the Extreme Ultraviolet Imaging Spectrometer (EIS) onboard Hinode. Magnetic field extrapolations suggest that the upflow regions seen in EIS were the sources of the outflowing solar wind observed with Metis. We find that the plasma associated with the narrow open-field corridor has higher electron densities and lower outflow velocities compared to the coronal hole plasma in the middle corona, even though the plasma properties of the two source regions in the low corona are found to be relatively similar. The speed of the solar wind from the open-field corridor also shows no correlation with the magnetic field expansion factor, unlike the coronal hole. These pronounced differences at higher altitudes may arise from the dynamic nature of the low-middle corona, in which reconnection can readily occur and may play an important role in driving solar wind variability.

**Supplementary Information:**

The online version contains supplementary material available at 10.1007/s11207-025-02438-8.

## Introduction

The solar corona continuously expands into interplanetary space and releases streams of plasma and magnetic field in the form of solar wind. Traditionally, the solar wind has been classified by its speed: the fast ($v$ > 450 km s^−1^) and slow ($v$ < 450 km s^−1^) solar wind. In situ measurements have revealed that these two types of solar wind have distinct properties. The fast solar wind generally has a lower plasma density, higher Alfvénicity and lower charge state ratio, implying a lower electron temperature at the source regions than its slower counterpart (Geiss, Gloeckler, and von Steiger 1995). The fast wind also does not have a significant enhancement in the abundance of elements with a low first-ionisation potential (FIP; Laming 2015), which is in contrast to the enhancement commonly found in the slow solar wind. It is also worth noting that slow solar wind streams exhibit more variability in their properties, with some portions even behaving like the fast solar wind (i.e. slow Alfvénic solar wind D’Amicis and Bruno 2015; Stansby et al. 2020; Yardley et al. 2024).

Despite the fact that solar wind streams have been studied for several decades, their origin and acceleration mechanisms still remain among the major open questions in heliophysics (e.g. Viall and Borovsky 2020). The fast solar wind is generally accepted to originate from the core of coronal holes (CHs), regions of the solar corona with relatively low plasma density and temperature, appearing dark in extreme ultraviolet (EUV) and soft X-ray images. The origin of the slow solar wind, on the other hand, is still under intense debate (e.g. Abbo et al. 2016). Although the slow Alfvénic wind is thought to originate from small, low-latitude CH or CH boundaries (Wang and Ko 2019; D’Amicis et al. 2021), the more variable slow solar wind, characterised by low-FIP element enhancements and higher charge state ratios, is argued to originate from hotter coronal regions, such as at the peripheries of active regions (ARs), where widespread plasma upflows are evident (Sakao et al. 2007; Brooks and Warren 2011; Brooks, Ugarte-Urra, and Warren 2015). Off-limb observations show that the slow solar wind emerges from extended coronal streamers observed in visible light, either from the open field at the streamer edges (Susino et al. 2008; Abbo et al. 2010) or as blobs from reconnection at the streamer cusps (Einaudi et al. 1999; Sheeley et al. 2009). The variable nature of the solar wind can also arise from rapid changes in magnetic connectivity to multiple source regions with different properties (Yardley et al. 2024).

Interchange reconnection between open and closed magnetic field lines has been suggested to be a mechanism for the release of solar wind plasma (Crooker, Gosling, and Kahler 2002; Fisk 2003). This process allows plasma trapped in closed field regions, such as ARs or coronal streamers, to be released along the (newly formed) open field, which may explain the coronal compositional signature of the slow solar wind (Baker et al. 2009; van Driel-Gesztelyi et al. 2012; Brooks, Ugarte-Urra, and Warren 2015; Yardley et al. 2024). Interchange reconnection may also be responsible for the release of the fast solar wind, as shown by the observation of small-scale coronal jets in CHs (Chitta et al. 2023b; Raouafi et al. 2023; Long et al. 2023) and the magnetic field reversals found in the solar wind in the inner heliosphere (Bale et al. 2019, 2023).

In particular, Antiochos et al. (2011) proposed that large-scale interchange reconnection takes place at the boundaries between closed- and open-field regions, which can be mapped to the network of separatrix surfaces and quasi-separatrix layers (QSLs; Demoulin et al. 1996) called the S-web. The S-web is theoretically defined as a set of arcs with drastic changes in magnetic connectivity found throughout the solar corona. The photospheric footpoints of the S-web structures are located at the boundaries of CHs, the peripheries of ARs and narrow, sometimes infinitesimal, open-field corridors that link disconnected CHs (Titov et al. 2011; Higginson et al. 2017; Scott, Pontin, and Wyper 2019). Structures such as helmet streamers or pseudostreamers are also intrinsically related to the S-web because they naturally give rise to open–closed field boundaries and magnetic null points (Wang, Sheeley, and Rich 2007; Titov et al. 2011; Antiochos et al. 2011).

Recently, Chitta et al. (2023a) presented observations of complex elongated plasma features in the off-limb EUV and visible light observations. These elongated plasma structures were interpreted as the imprints of the S-web above the CH-AR system and also as observational evidence of the slow solar wind streams. Baker et al. (2023) investigated plasma upflows associated with a thin open-field corridor embedded in an AR, which was then associated through magnetic connectivity mapping with variable slow solar wind streams. These results were interpreted as supporting evidence of the framework that reconnection dynamics along the S-web are responsible for the release of (at least part of) slow solar wind plasma.

The acceleration of the solar wind, as well as the transition from a closed- to an open-field configuration, occur in the middle corona, here defined as the region with a heliocentric distance of 1.5 – 6 R_⊙_ (West et al. 2023). Previous coronagraph observations showed significant differences in the electron density and solar wind outflow speeds in equatorial coronal streamers compared to polar CHs in the middle corona, with polar CHs having lower densities and higher solar wind speeds (Antonucci et al. 2004; Antonucci, Abbo, and Dodero 2005; Abbo et al. 2010). The outflow speeds seem to correspond to the degree of super-radial expansion of open magnetic flux tubes (Wang and Sheeley 1990). In addition, preferential heating and acceleration of the ions are observed in both the fast solar wind from CHs (Cranmer, Panasyuk, and Kohl 2008) and (to a lesser extent) the slow solar wind from streamers (Spadaro et al. 2007; Abbo et al. 2010), suggesting the effect of kinetic-scale physics, such as ion cyclotron resonant waves (see reviews by, e.g. Antonucci 2006; Cranmer 2009; Cranmer and Winebarger 2019).

The complex and dynamic nature of the corona has been revealed by high spatiotemporal off-limb observations. The fine-scale structures of coronal plasma and magnetic field in the low corona can be identified from total solar eclipse observations in visible light (Habbal et al. 2011, 2021; Druckmüller, Habbal, and Morgan 2014), as well as EUV imaging (Seaton et al. 2021; Morton and Cunningham 2023). Higher up in the middle and extended corona, similar structures are found to constantly propagate outward from the Sun and impose themselves in the interplanetary solar wind, as shown by coronagraph observations (DeForest et al. 2018; Alzate et al. 2021). The density variations and reconfigurations of these fine-scale structures may imply that ongoing reconnection is taking place, which can also contribute to the energisation and acceleration of the solar wind (Chitta et al. 2023a; Liewer et al. 2023; Ventura et al. 2023).

In this paper, we attempt to provide new insights into the origin of the solar wind using coordinated remote sensing observations between the Solar Orbiter (SO; Müller et al. 2020) spacecraft and Earth-orbiting satellites, namely, the Solar Dynamics Observatory (SDO; Pesnell, Thompson, and Chamberlin 2012) and Hinode (Kosugi et al. 2007). We simultaneously derive properties of solar wind plasma emanating from two open-field regions in the low corona (using on-disk spectroscopy) and in the middle corona (using off-limb coronagraph observations), which we link together using magnetic extrapolations. The paper is structured as follows. The instruments and datasets used in this study are described in Section 2. The magnetic extrapolation methods and the global magnetic structure of the solar corona are detailed in Section 3. In Sections 4 and 5, we present the main results from the analysis of the solar wind in the low coronal and the middle coronal observations, respectively. Section 6 shows the evolution and dynamics of the low and middle corona. Finally, we discuss the results and summarise our findings in Section 7.

## Remote Sensing Observations

On 2023 April 9, Solar Orbiter reached perihelion at a heliocentric distance of 0.29 AU and was located around 60^∘^ west of the Sun–Earth line. This configuration made it possible to observe the solar corona using remote sensing instruments from two different viewpoints: Earth-based satellites (SDO and Hinode) and SO. Note that the difference in distances of the individual spacecraft from the Sun meant that phenomena were observed at different local spacecraft times. Hence, to avoid confusion, we will use the time at Earth for all observations in this paper.

We investigated the solar wind outflows in the middle corona using the Metis coronagraph (Antonucci et al. 2020a; Fineschi et al. 2020) onboard SO. Metis provides coronagraph observations of the off-limb corona in an annular field of view (FOV) ranging 1.6 – 2.9^∘^ (corresponding to 1.76 – 3.75 R_⊙_ at a heliocentric distance of 0.29 AU) in two passbands: visible light (VL; 580 – 640 nm) and ultraviolet neutral hydrogen Ly$\alpha $ (UV; $121.6 \pm 10$ nm). From 04:55 UT to 23:55 UT, Metis acquired sequences of VL total brightness (tB) and polarised brightness (pB) images in parallel with UV Ly$\alpha $ images, with a temporal cadence of 20 min, a total effective exposure time of 15 min for both channels and spatial resolutions of 20^′′^ per pixel in the VL and 40^′′^ per pixel in the UV. We used level 2 data, which were calibrated using the most up-to-date calibration available and can be accessed through the Solar Orbiter Archive (SOAR).^1^ Standard calibration operations include correction for detector bias, dark current, and flat field, optical vignetting function and radiometric calibration (De Leo et al. 2023, 2024).

We also used extreme-ultraviolet (EUV) images in the 174-Å passband from the Full Sun Imager (FSI) telescope of the Extreme Ultraviolet Imager (EUI; Rochus et al. 2020) onboard SO to investigate the low-coronal structure in the region below Metis’ inner FOV and complement middle corona observations. This telescope takes full solar disc images with a plate scale of 4.4^′′^ per pixel and a time cadence of 10 min. We used calibrated level 2 EUI FITS files from EUI data release 6 (Kraaikamp et al. 2023) for this analysis.

From Earth’s viewpoint, we investigated the plasma dynamics in the regions directly below Metis’s FOV. As detailed in Hinode Operation Plan 462,^2^ the EUV Imaging Spectrometer (EIS; Culhane et al. 2007) onboard the Hinode satellite constructed two consecutive column mosaics approximately 30^∘^ east of the central meridian that correspond to the east solar limb as seen by SO. In this observing campaign, two EIS studies were used. The first study was DHB_007_v2, a raster scan with a FOV size of $248^{\prime\prime} \times 512^{\prime\prime}$. The slit size was 2^′′^ with an exposure time of 60 s and rastered in 4^′′^ steps. The second study used was CH_bound_240x512v1, a raster scan with a FOV size of $240^{\prime\prime}\times 512^{\prime\prime}$. The slit size was also 2^′′^ and rastered in 4^′′^ steps, but each slit had an exposure time of 100 s.

Two of the 12 total scans were used for the analysis in this work. The first scan used the DHB_007_v2 study and was run from 07:11 UT to 08:15 UT centred at (x, y) ∼ (-473^′′^, -607^′′^). The second scan used the CH_bound_240x512v1 study centred at (x, y) ∼ (-475^′′^, 617^′′^) and was run in the period 12:13 UT–13:55 UT. The obtained spectra were corrected for instrumental effects, including slit tilt, orbital variation, dark current and warm/hot/dusty pixels before further analysis. Plasma dynamics and properties were then obtained by fitting the spectral data using the EISPAC Python library (Weberg et al. 2023).

Lastly, to provide context for the overall structure and investigate magnetic properties of the solar wind source regions, we used observations from the Atmospheric Imaging Assembly (AIA; Lemen et al. 2012) and the Helioseismic and Magnetic Imager (HMI; Scherrer et al. 2012) onboard SDO. AIA continuously monitors the full solar disk in seven EUV passbands, with a plate scale of 0.6^′′^ per pixel and 12 s cadence. On the other hand, HMI provides line-of-sight (LOS) photospheric magnetograms with a temporal resolution of 45 s and a plate scale resolution of 0.505^′′^ per pixel. The level 1 AIA observations were processed using a standard routine in the aiapy Python library (Barnes et al. 2020). The HMI magnetograms were then coaligned with the AIA observations.

**Figure 1:**
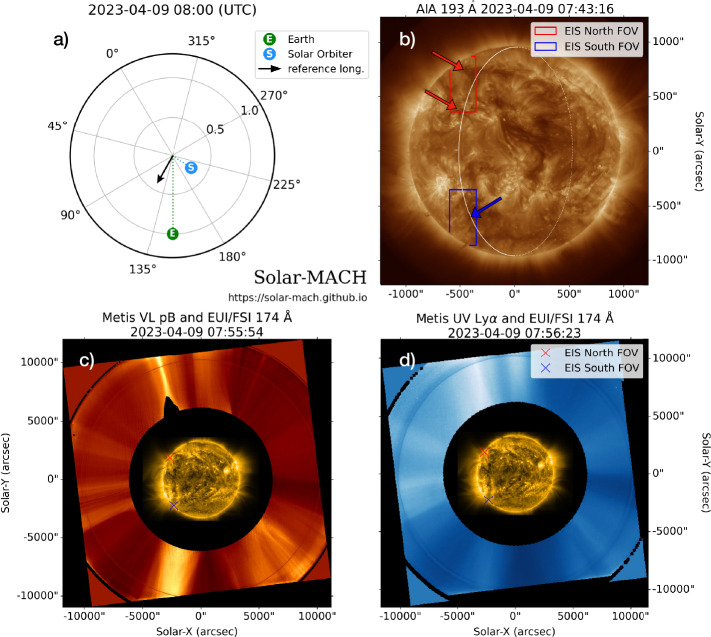
Overview of the remote sensing observations made by SO, Hinode and SDO on 2023 April 9. (a) The diagram showing position of Earth (equivalent to SDO and Hinode) and SO in Carrington coordinate system. The black arrow indicate the Carrington longitude of the east solar limb seen by SO. (b) AIA 193 Å image with the solar limb seen by SO is plotted as a white line. The red arrows point to the solar filaments, and the blue arrow points to a small CH. (c) The composite of EUI/FSI 174 Å and Metis VL pB observations. (d) The composite of EUI/FSI 174 Å and Metis UV Ly$\alpha $ observations. The EIS FOVs are shown in coloured boxes and crosses, red for the North FOV and blue for the South FOV.

Figure 1 provides an overview of the remote sensing observations we used for this analysis. Panel a shows the top view of the position of Earth and SO during the observation period generated from the SOLAR-MACH tool (Gieseler et al. 2023), indicating that SO was located at $\sim 60^{\circ}$ west of the Sun–Earth line, and the east limb (black arrow) seen from SO can be observed on the solar disc by Earth-orbiting Hinode and SDO.

Panel b displays the solar corona observed from Earth’s perspective using AIA. The FOVs of two EIS raster scans are shown in the red and blue boxes in the left panel, which partly overlap the location of the solar limb as seen by SO, denoted as a white line. The EIS South FOV (blue) corresponds to a small mid-latitude coronal hole (CH; blue arrow), which was identified as a relatively dark region on the solar disc in AIA 193 Å observations. The EIS North FOV (red), on the other hand, observes a relatively bright region lying between two solar filaments, denoted by two red arrows. We interpret that this region corresponds to a decayed AR because NOAA AR 12331 was observed at the same exact location on 2023 February 14, two solar rotations prior to our observations.

Panels c and d present the observations of the low and middle corona by EUI and Metis onboard SO. The pB and UV observations are enhanced using the Normalising-Radial-Graded Filtering method (NRGF; Morgan, Habbal, and Woo 2006). The red and blue crosses correspond to the centre of the EIS North and South FOVs, confirming that they were observing the east solar limb region seen from SO as planned. The middle corona region corresponding to the EIS South FOV has a lower emission in the VL pB and UV Ly$\alpha $ intensity than that above the EIS North FOV, as observed from Metis.

## Global Magnetic Configuration

The structure and plasma dynamics in the low and middle corona are directly related to the global magnetic field configuration. Therefore, we employ a magnetic field extrapolation based on Predictive Science’s Magnetohydrodynamics Around a Sphere simulation code (PSI-MAS; Mikić et al. 1999) for this analysis. This method solves a set of resistive magnetohydrodynamics (MHD) equations in a 3D non-uniform spherical coordinate grid covering a heliocentric distance of $1 - 30~\mathrm{R}_{\odot}$ to simulate the volume-filling plasma and magnetic field states of the solar corona. A synoptic photospheric magnetogram is used to provide the boundary condition for the radial magnetic field at 1 R_⊙_. Various known processes in the solar corona, such as radiative losses, anisotropic thermal conduction and coronal heating, have also been accounted for in the simulation with several degrees of sophistication (Riley et al. 2021). The PSI-MAS model is generally consistent with total solar eclipse observations (e.g. Boe et al. 2021; Boe, Downs, and Habbal 2023), which demonstrates that the model can reasonably predict the structure in the low and middle corona. A more detailed description of the PSI-MAS model can be found in Mikić et al. (1999), Lionello, Linker, and Mikić (2009) and Mikić et al. (2018).

In our analysis, we used the semiempirical thermodynamic model of the PSI-MAS simulation described in Lionello, Linker, and Mikić (2009), which accounts for the energy transport processes and plasma dynamics using an empirical heating function. The HMI radial synoptic map from Carrington rotation 2269 (CR2269; 2023 March 24–April 20) is chosen as the lower boundary condition. The simulation results are publicly available from Predictive Science’s website,^3^ which includes the three components of the magnetic field and several important plasma properties. We traced magnetic field lines from the photosphere up to 30 R_⊙_, making it possible for us to investigate magnetic field structures without common constraints such as the source surface height.

**Figure 2:**
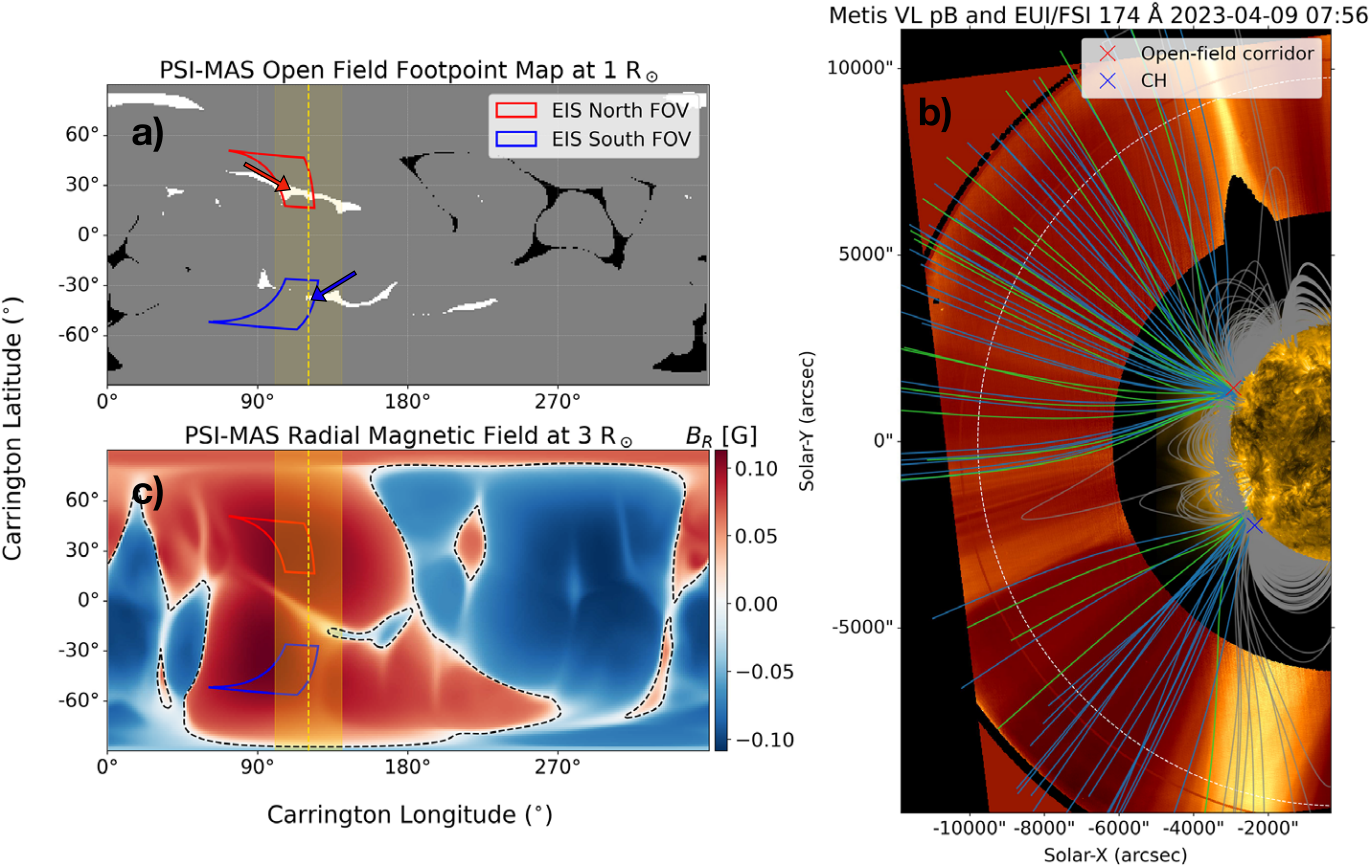
The magnetic field extrapolations derived from the PSI-MAS MHD simulation during Carrington rotation 2269. (a) Carrington synoptic map showing the footpoints of the positive (white) and negative (black) polarity of the open field. The yellow-shaded region indicates the area corresponding to the east solar limb seen by Metis, from where the field lines in panel b) were traced. The red and blue arrows point to the open-field footpoints corresponding to a thin open-field corridor and a small CH, respectively. (b) Extrapolated magnetic field lines plotted over the composite EUI/FSI 174 Å and Metis VL pB observations. The grey lines indicate the closed-field lines. The light green lines highlight the open-field lines with an expansion factor $f_{s}$ > 20, and the blue field lines show those with $f_{s}$ < 20 (see text). The red and blue crosses denote the location of the open-field corridor and the CH. The dashed white line denotes a heliocentric distance of 3 R_⊙_. (c) Carrington synoptic map of the radial magnetic field at 3 R_⊙_. Positive (negative) polarities are shown in red (blue). The polarity inversion lines separating positive and negative polarities are indicated by black dashed lines, with the longest line corresponding to the heliospheric current sheet.

Figure 2 summarises the results from the magnetic field extrapolations. Panel a shows the locations of the open field at the photosphere for CR2269. The east limb as seen by Metis and surrounding regions ($\pm 20^{\circ}$) are denoted as yellow dashed lines and shaded regions. The locations of EIS’s FOVs (red and blue boxes) overlap with the footpoints of positive-polarity open magnetic fields (white regions). In particular, the EIS North FOV corresponds to a thin open-field corridor at Carrington latitude $\sim 30 ^{\circ}$, indicated by a red arrow. The EIS South FOV, on the other hand, corresponds to the CH, indicated by a blue arrow (see also Figure 1). This strongly suggests that EIS was observing the source regions of the solar wind. Hence, if there are any plasma upflows along these open-field regions observed by EIS, they are likely to be the low-coronal origin of the solar wind.

Panel b of Figure 2 illustrates the selected extrapolated field lines overlaid on the composite Metis VL pB and EUI/FSI 174 Å observations. All plotted field lines have footpoints within the yellow-shaded region in panels a and c. Near the equator, we can see that there are closed magnetic fields associated with a large pseudostreamer. This structure is surrounded by two open-field regions in the north and south, which are rooted in the open-field footpoints inside the FOVs of the EIS observations, i.e. the open-field corridor (red cross) and the CH (blue cross). The extrapolated magnetic field lines match well with the structure of the middle corona observed by Metis. In particular, the open-field structure in the south corresponds to the dark region seen in Metis pB observations, associated with a CH region. The northern open-field region, on the other hand, appears brighter in pB observations compared to the region corresponding to the southern CH.

Panel c shows the derived radial magnetic field strength in the middle corona at 3 R_⊙_. At this height, the east solar limb seen by SO is mainly filled by positive open magnetic fields that expand from the photosphere. The heliospheric current sheet, defined as the boundary between positive and negative open field regions, is highly inclined (shown by the longest black dashed line), indicating that the solar magnetic field configuration during this time is much more complex compared to the simple dipolar field during solar minimum. The helmet streamer near the south pole seen in panel b also roughly marks the location of the heliospheric current sheet.

We then quantify the properties of the coronal magnetic field using two parameters. The first parameter is the expansion factor which measures the degree of super-radial expansion of open magnetic flux tubes. The expansion factor $f_{s}$ is defined as (Wang and Sheeley 1990; Antonucci et al. 2023)

1 $$ f_{s} = \left (\frac{\text{R}_{\odot}}{r_{1}} \right )^{2} \frac{B_{R}(\text{R}_{\odot})}{B_{R}(r_{1})} , $$

where $B_{R}(\text{R}_{\odot})$ and $B_{R}(r_{1})$ are the radial magnetic field strengths at the photosphere and at a specific radial distance $r_{1}$ in the corona. Using the potential field source surface extrapolations and the measured solar wind speed at 1 AU, Wang and Sheeley (1990) found an inverse correlation between the solar wind speed and $f_{s}$, where $f_{s}$ is computed at $r_{1} = 2.5~{\mathrm{R}}_{\odot}$ (that is, the radius of the source surface). In general, low expansion factors correspond to fast solar wind speeds, whereas high expansion factors correspond to slow solar wind speeds. We illustrate the expansion factors associated with open-field lines in panel b of Figure 2. The light green lines indicate open-field lines with $f_{s}$ greater than 20 at $r_{1} = 3~\mathrm{R}_{ \odot}$, while the blue lines indicate those with $f_{s}$ less than 20. This criterion is similar to that defined in Wang and Ko (2019).

Another parameter called the squashing factor Q (Titov 2007) is also computed to better characterise the boundaries between the open and closed magnetic field structures. In general, Q measures the deformation of circular magnetic flux tubes in the photosphere into elliptic flux tubes in the corona, which in turn quantifies the divergence of local magnetic field lines and gradients in field-line mapping. Hence, the Q value is large at the boundaries of two different magnetic topologies, such as at separatrix surfaces ($\mathrm{Q}\rightarrow \infty $) or at quasi-separatrix layers (QSLs) (Q > 10^3^; Antiochos et al. 2011). The high Q regions, in which field lines from different magnetic domains converge, may be preferable sites for magnetic reconnection processes. In this analysis, we calculated Q from the coordinates and magnetic field values obtained from the results of the PSI-MAS simulation discussed earlier in this section. For each point on the surface, we defined flux tubes by tracing the field lines forwards and backwards five times (from the point itself and four neighbouring points). We then estimated Q from the coordinates and magnetic field properties at the boundaries of the flux tubes (PSI 2024).

**Figure 3:**
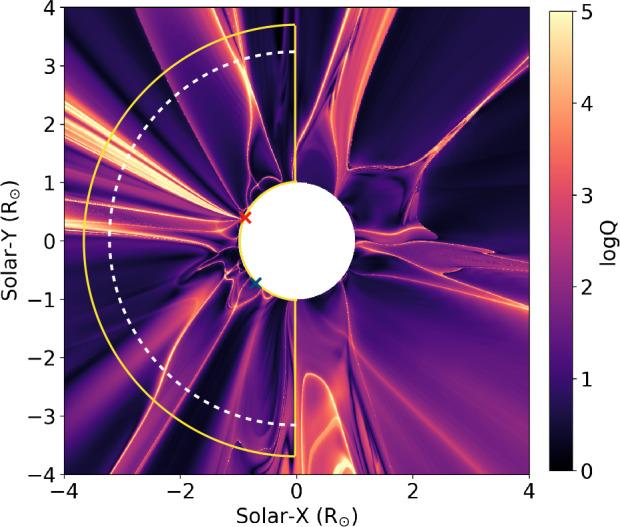
A map of squashing factor (logQ) values showing the magnetic structures of the low-to-middle corona as seen from SO on 2023 April 9. The red and blue crosses mark the location of two FOVs of EIS. The yellow contour illustrates the solar east limb region that corresponds to the polar projection in Figure 9, and the white dashed line marks the heliocentric distance of 3 R_⊙_.

Figure 3 shows the distribution of logQ values in the plane of the sky (POS) as seen from Metis during the observation period. The region above the EIS North FOV (red cross) consists of several complex high Q arcs (logQ > 3), particularly near the equatorial region. However, the region above the EIS South FOV generally has lower Q values with only a few high Q arcs. Hence, this further highlights the different magnetic environments in the middle corona above two different source regions of the solar wind.

## Low Corona Plasma Diagnostic—SDO and EIS

### Overview of the Solar Wind Source Regions

**Figure 4:**
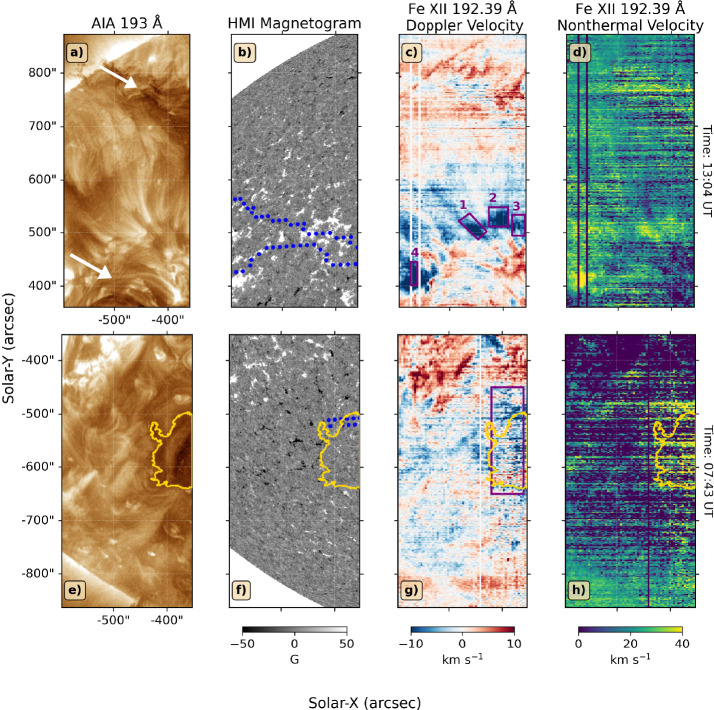
Plasma diagnostics of the low solar corona at the open-field corridor (EIS North FOV, top row) and CH (EIS South FOV, bottom row). From left to right, the columns show AIA 193-Å observations, HMI LOS photospheric magnetograms, EIS Fe xii 192.39 Å Doppler velocity maps and EIS Fe xii 192.39 Å nonthermal velocity maps. The yellow contours indicate the CH boundaries, and the dotted blue contours outline the open-field locations. The purple boxes indicate the regions where we average the spectra to obtain plasma properties shown in Table 1. The colour scale of the Doppler velocity maps was set to [-10,10] km s^−1^. The nonthermal velocity maps are saturated at [0,40] km s^−1^. HMI magnetograms are saturated at $\pm 50$ G.

The solar wind source regions in the low corona were observed by the AIA and HMI instruments onboard SDO and the EIS spectrometer onboard Hinode. Figure 4 shows an overview of the low coronal observations at the EIS North FOV (top row) and the South FOV (bottom row). Each row shows the AIA 193 Å observations enhanced using the Multiscale Gaussian Normalisation technique (MGN; Morgan and Druckmüller 2014), the HMI LOS magnetogram and the Doppler velocity and nonthermal velocity maps obtained from fitting the EIS Fe xii 192.39 Å spectral line. From panels a and e, the EIS North FOV corresponds to the decayed AR 12331 between two solar filaments (pointed by two white arrows), whereas the EIS South FOV corresponds to the eastern boundary of a small mid-latitude CH. The CH boundaries are defined using an intensity thresholding technique, with the threshold chosen to be 40% of the median solar disc intensity in the 193-Å passband (Heinemann et al. 2019). The boundaries are plotted in the yellow contours seen in the bottom row of Figure 4.

Panels b and f of Figure 4 show the photospheric magnetic field strength in the northern and southern regions, with the location of the open-field footpoints derived from the PSI-MAS model (see also Figure 2) denoted in dotted blue contours. This confirms that parts of the regions observed inside EIS’s FOVs were magnetically open. Panel b also shows that the quiet Sun region observed in the EIS North FOV corresponds to a thin, latitudinal, open-field corridor at the base of the pseudostreamer (see panel a of Figure 2). Both footpoint locations correspond to the positive-polarity magnetic field, with the open-field corridor being associated with a stronger field than the CH counterpart.

Note that the locations of open-field footpoints derived from the PSI-MAS model do not exactly match the locations of CH derived from EUV observations. The open-field corridor does not correspond to relatively dark regions (i.e. CH) on the solar disc. Instead, the corresponding region seems to consist of several bright plumes. The area of the CH in the EIS south FOV is also considerably larger than the open-field region derived from the model, which only captures the northern boundary of the CH. This mismatch may result from the limitation of magnetic modelling and photospheric magnetic field observations. Note that the disagreement between the area of open flux regions in the model and EUV observations is part of the so-called ‘Open Flux Problem’ (Linker et al. 2017; Asvestari et al. 2024).

Doppler and nonthermal velocity maps can reveal the dynamics of plasma in the low corona. For the open-field corridor, plasma upflows, indicated by the blue-shifted region on the Doppler velocity map, were observed throughout the full longitudinal extent of EIS’s FOV and spanning from 400^′′^ to 600^′′^ in helioprojective latitude (see panel c of Figure 4). The upflow region appears to have a fan-like structure similar to the upflows seen at the edge of active regions (Brooks, Ugarte-Urra, and Warren 2015; Yardley, Brooks, and Baker 2021; Baker et al. 2023). The base of the upflows has enhanced nonthermal velocities (see panel d), which also roughly correspond to the region with strong positive magnetic flux. The location of the upflows also coincides with the thin open-field corridor derived from the PSI-MAS model and the plume-like structures seen in the AIA 193 Å passband. Hence, our interpretation is that the upflowing plasma travels upwards along the open-field lines, becoming the outflowing solar wind.

We also found upflow regions within the southern CH and the surrounding boundary region. However, the upflow locations are more dispersed throughout the CH area (see panel g). There is also less enhancement in the nonthermal velocity compared to the north region. Since CHs are well-known to have open magnetic field configurations, the plasma upflows inside CH are also thought to form part of the outflowing solar wind.

### Plasma and Magnetic Properties of Upflow Regions

To quantitatively analyse the plasma parameters of these upflow regions, we defined several boxes based on the locations of strong upflows: four boxes in the EIS North FOV focusing on the regions with strong upflows in the open-field corridor and one box in the South FOV covering the eastern boundary of CH. The locations of these boxes are denoted as purple boxes in panels c and g of Figure 4. Plasma parameters were then derived using the averaged spectra of the pixels confined in each box to enhance the signal-to-noise ratio of the data .^4^

Table 1 shows the plasma and magnetic field measurements inside each box. Density measurements were derived using the spectral intensity ratio between Fe xiii 203.83 Å and 202.04 Å. The first ionisation potential (FIP) bias values were calculated using the diagnostic of the lines pair Si x 258.37 Å and – S x 264.22 Å following the method of Brooks and Warren (2011). The uncertainties are $\sim30\%$ for log_10_ density and FIP bias value, $\sim5$ km s^−1^ for Doppler velocity and $\sim20$ km s^−1^ for nonthermal velocity. Assuming the magnetic field is radial, we also reprojected the observed line-of-sight magnetic field component for each magnetogram pixel (Hofmeister et al. 2017) before calculating the mean magnetic flux density inside each box.

**Table 1:** Plasma and magnetic properties inside the regions of interest denoted by purple boxes in panel c and g in Figure 4.

Location (Raster time)	Open-Field Corridor (13:04 UT)				CH (07:43 UT)
Box No.	1	2	3	4	-
Mean Magnetic Flux Density (G)	45.4	39.2	21.5	34.7	1.0
Log_10_ Density (cm^−3^)	8.2	8.3	8.3	8.3	8.3
FIP Bias	0.8	1.1	1.0	1.1	1.3
Doppler Velocity (km s^−1^)	-6	-14	-9	-8	-5
Nonthermal Velocity (km s^−1^)	27	28	27	29	31
Secondary Component	Yes	No	No	Yes	No

In all boxes, we find that the plasma density and FIP bias values are very similar, with the density values in the range of $\approx 10^{8.2}$ – $10^{8.3}$ cm^−3^ and the FIP bias values between 0.8 and 1.3. These values correspond to those that typically characterise CH plasma, which has a relatively low density (Hahn et al. 2010; Heinemann et al. 2021) and little to no enhancement of the low FIP elements (i.e. FIP bias ∼ 1, Feldman et al. 1998; Brooks and Warren 2011).

The plasma dynamics in each box are also quite similar. For the open-field corridor (Boxes 1 – 4), the Doppler velocity ranges from -6 to -14 km s^−1^ and the nonthermal velocity ranges from 27 to 29 km s^−1^. For the CH region, the Doppler and nonthermal velocity values are -5 km s^−1^ and 31 km s^−1^. Note that the lower values may be the result of using relatively large macropixel boxes, which may average the strong upflows with surrounding weaker upflow regions.

The most striking differences between the open-field corridor and the CH are the magnetic field properties. For the open-field corridor, the average magnetic flux density values range from 21 to 45 G, which are comparable to the values of decayed ARs (Petrie and Haislmaier 2013) and the narrow AR upflow corridor (Baker et al. 2023). The mean magnetic flux density of the small CH is 1 G, which is similar to the typical values in nonpolar CHs (≈ 1 – 5 G, Hofmeister et al. 2017; Heinemann et al. 2019) and significantly lower than the values in the open-field corridor.

**Figure 5:**
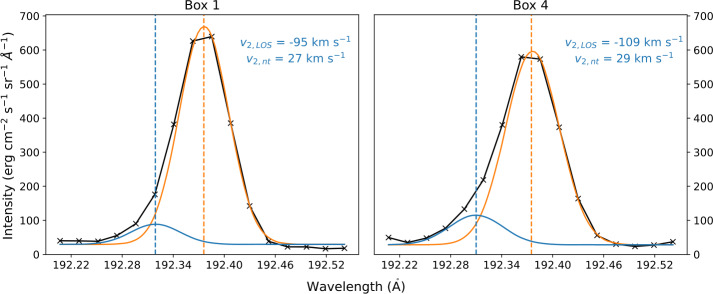
Fe xii 192.39 Å averaged spectral line profiles obtained from Box 1 (left) and Box 4 (right), both correspond to the open-field corridor region. Both profiles can be fitted using the double Gaussian function, indicating the existence of the weak emission, high-speed secondary component of plasma upflow (blue). The calculated LOS velocity and nonthermal velocity of the secondary component are shown in each panel.

The averaged Fe xii 192.39 Å spectral line profiles in Box 1 and Box 4 show significant blue-wing enhancements, suggesting that there is at least one secondary component of high-speed plasma upflows. To analyse these features, we used a double Gaussian function (Brooks and Warren 2012; Yardley, Brooks, and Baker 2021; Ngampoopun et al. 2023) to fit both line profiles and derived the plasma dynamics of secondary upflows, as shown in Figure 5. In both profiles, significant secondary components are found, that is, the intensity of the secondary component is more than 10% of the primary component. The LOS velocity of the secondary components is -95 km s^−1^ for Box 1 and -109 km s^−1^ for Box 4, which are comparable to the high-speed components of AR upflows (Tian et al. 2021). Since we choose to fit the double Gaussian with the same width, the nonthermal velocities of the secondary components are the same as their primary components counterpart, which is a reasonable assumption (Tian et al. 2011). These high-speed upflows are frequently observed near the edges of ARs (e.g. Hara et al. 2008; Brooks and Warren 2012; Yardley, Brooks, and Baker 2021) and in coronal jets arising from open-field regions (Young and Muglach 2014; Ngampoopun et al. 2023).

## Middle Corona Plasma Diagnostic—Metis

The properties of solar wind streams in the middle corona can be investigated using VL and UV coronagraph observations made by Metis. The two main properties that can be derived from these observations are the distributions of electron density and the outflow velocity of neutral hydrogen (H i) in the corona.

### Electron Density

The observed polarised brightness (pB) in the VL passband mainly arises from Thomson scattering between photospheric photons and free electrons in the corona. Although light from interplanetary dust scattering may be slightly polarised (Morgan and Cook 2020; Boe et al. 2021), pB emission from regions close to the Sun is still dominated by scattered light from electrons (e.g. Lamy et al. 2020). Therefore, the electron density in the middle corona can be determined on the basis of the pB distribution. The relationship between pB and electron density $n_{e}(r)$ is as follows (van de Hulst 1950; Hayes, Vourlidas, and Howard 2001):

2 $$ pB = C\int _{x}^{\infty} n_{e}(r)[A(r) - B(r)] \frac{x^{2} dr}{r\sqrt{r^{2} - x^{2}}} ,$$

where $A(r)$ and $B(r)$ are geometrical factors, $C = 3.44 \times 10^{-6}$ cm^−3^ is a unit conversion factor, $x$ is the projected distance on the POS and $r$ is the heliocentric distance.

In this analysis, we follow the pB inversion method described by van de Hulst (1950) and Hayes, Vourlidas, and Howard (2001). This method derives the electron density on the basis of two assumptions that the distribution of electron density is axisymmetric along the axis of solar rotation, and the density profile can be expressed in the polynomial form

3 $$ n_{e}(r) = \sum _{k} (\alpha _{k} r^{-k}) , $$

where $k$ is the polynomial degree and $\alpha _{k}$ is the unknown coefficient. By substituting the polynomial form into Eq. 2, $\alpha _{k}$ can be solved using the multivariate least-squares fitting method. For our calculation, we perform the fit for pB observations in the range of $r = 1.8 - 3.5~\mathrm{R}_{\odot}$ to avoid noisy pixels close to the inner and outer FOV of the instrument. We also found that using $k = (1 - 5)$ provided the best fitting results. The uncertainties are estimated to be approximately 10% (e.g. Dolei et al. 2018; Romoli et al. 2021).

**Figure 6:**
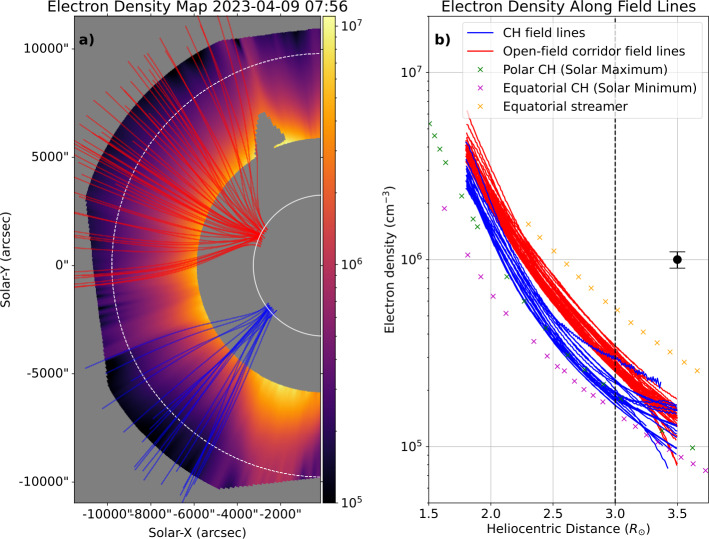
Electron density derived from pB inversion method. (a) Electron density map of the solar east limb seen by Metis, with overplotted extrapolated open-field lines from the open-field corridor (red lines) and CH (blue lines). (b) Plot of electron density along the open-field lines (red for the open-field corridor and blue for the CH) against distance from the Sun’s centre. The coloured crosses indicate the reported values of the electron density from equatorial CHs during solar minimum (purple; Withbroe 1988), polar CHs during solar maximum (green; Withbroe 1988), and equatorial streamers (orange; Hayes, Vourlidas, and Howard 2001). The white and black dashed lines indicate the heliocentric distance $r = 3~\mathrm{R}_{\odot}$. The representative error of $\approx \pm 10 \%$ is indicated as a black error bar.

Figure 6 shows the electron density resulting from the inversion of Metis VL pB observations at 07:56 UT. Panel a shows the 2D electron density map of the solar east limb as seen by Metis, which depicts the overall structure of the middle corona. During this period, the east limb consisted of a high-density streamer in the equatorial region and lower-density open-field regions surrounding it. The blue and red field lines denote the open fields rooted in the CH and the open-field corridor, respectively. The open field lines from both regions are derived from the PSI-MAS model as discussed in Section 3.

The electron density values along these open field lines projected on the POS as a function of the heliocentric distance are displayed in panel b of Figure 6. In general, the electron density decreases by more than an order of magnitude as the heliocentric distance increases from 1.8 to 3.5 R_⊙_. The electron density of the CH plasma (blue lines) is similar to the polar CH density at solar maximum and considerably higher than the equatorial CH density at solar minimum reported in Withbroe (1988) (green and purple crosses). The electron density in the open-field corridor (red lines) is lower than the density in the equatorial streamers derived by Hayes, Vourlidas, and Howard (2001) (orange crosses), but it is still noticeably higher than the CH plasma. The differences become more apparent at $r$ > 2.3 R_⊙_, where the open-field corridor plasma has the density of $\approx 1.1 \times 10^{6} \ \text{cm}^{-3}$ and the CH plasma has the density of $\approx 0.7 \times 10^{6} \ \text{cm}^{-3}$. The differences also are visually evident on the electron density map.

### H I Outflow Velocity

The UV Ly$\alpha $ intensity observed in the off-limb corona is mainly due to resonance scattering of chromospheric Ly$\alpha $ radiation by coronal H i atoms (Gabriel 1971). The coupling between protons and H i atoms in the low-to-middle corona, caused by rapid charge transfers between them, allows us to use H i atoms as a proxy for protons (Allen, Habbal, and Hu 1998; Kohl et al. 2006). In the presence of the solar wind, the centroid of the incident chromospheric Ly$\alpha $ spectral line is Doppler-shifted from the centroid of the coronal H i absorption profile, resulting in a systematic and progressive reduction in scattered intensity called the Doppler dimming effect (Hyder and Lites 1970; Withbroe et al. 1982; Noci, Kohl, and Withbroe 1987). The intensity is increasingly reduced with higher outflow speeds up to 450 km s^−1^ (see Figure 1 in Dolei et al. 2018). Hence, we can exploit this effect to derive the outflow speed of H i atoms (equivalent to proton speed) in solar wind streams.

The derivation of coronal outflow speed based on the Doppler dimming effect involves creating the synthetic Ly$\alpha $ intensity based on several assumptions and input parameters. In particular, the outflow speed is treated as a free parameter in order to match the synthetic intensity with the observation made by the coronagraph. The method description and effects of the uncertainties of input parameters are extensively detailed in Dolei et al. (2018).

Important input parameters include the electron temperature (T_*e*_) and the hydrogen kinetic temperature (T_*k*_), which cannot be directly determined from available coronal observations (from Metis or other instruments). Therefore, we have to adopt temperature profiles derived from similar coronal structures from previous analyses reported in the literature. In particular, Gibson et al. (1999) obtained the temperature profile from visible-light observations of large helmet streamers during the solar minimum, assuming the hydrostatic equilibrium condition. In addition, Vásquez, van Ballegooijen, and Raymond (2003) derived the analytical form of the temperature profile in the polar and equatorial regions, which is in good agreement with observations from UV coronagraphs. Both models are frequently used in the full Sun Doppler dimming analysis based on Metis observations (e.g. Romoli et al. 2021; Antonucci et al. 2023). Another critical assumption is the degree of anisotropy in T_*k*_, defined as the ratio between kinetic temperature in the direction perpendicular (T_*k*,⊥_) and parallel (T_*k*,∥_) to the magnetic field. Polar CH regions have been reported to show a strong anisotropy in T_*k*_ (Cranmer et al. 1999), while equatorial regions typically show weaker anisotropy or isotropic conditions (Vásquez, van Ballegooijen, and Raymond 2003; Spadaro et al. 2007).

Since the coronal structures in our observations are quite complex as the Sun approaches solar maximum, we explored multiple cases using various temperature profiles relevant to both the equatorial and polar regions and various degrees of anisotropy to find reasonable assumptions that suit our observation. After careful consideration, we employed the following set of assumptions to create the synthetic Ly$\alpha $ intensity:

- electron density derived from inversion of Metis pB images, as discussed in Section 5.1
- chromospheric Ly$\alpha $ line profile from an analytical model by Auchère (2005)
- uniform chromospheric Ly$\alpha $ line intensity across the solar disc with $I_{\odot }= 7.86\times 10^{15}~\text{photon s}^{-1}~\text{cm}^{-2}~\text{sr}^{-1}$. The value is computed from the daily-averaged Ly$\alpha $ solar irradiance observed from Earth on the observation date, available at LASP Interactive Solar Irradiance Data Center^5^
- T_*e*_ radial profile based on the profile of polar regions described in Vásquez, van Ballegooijen, and Raymond (2003). The original profile is scaled so that the base temperature $\approx 1$ MK, corresponding to the typical coronal temperature in the mid-latitude regions.
- mild anisotropy ($\approx 2$) condition, with $\mathrm{T}_{k, \parallel} = \mathrm{T}_{e}$
- T_*k*,⊥_ radial profile based on the functional form given by Vásquez, van Ballegooijen, and Raymond (2003). The profile is then adjusted to ensure mild anisotropy condition (i.e. $\mathrm{T}_{k, \perp} \approx 2\mathrm{T}_{e}$) in the middle corona.
- integration of off-limb Ly$\alpha $ intensity along LOS of $\pm 10~\mathrm{R}_{\odot}$ from the plane of the sky.

To reduce some intensity fluctuations arising from an instrumental effect that affects the Metis UV detector (Russano et al. 2024; Uslenghi et al. 2024; De Leo et al. 2024), we averaged five Ly$\alpha $ images acquired between 07:16 UT and 08:36 UT before deriving the coronal wind outflow velocity with the Doppler dimming technique. Note that the uncertainty in the derived speed due to calibration and data uncertainties is estimated to be on the order of $\sim 20~\text{km}~\text{s}^{-1}$ (e.g. Antonucci et al. 2023).

**Figure 7:**
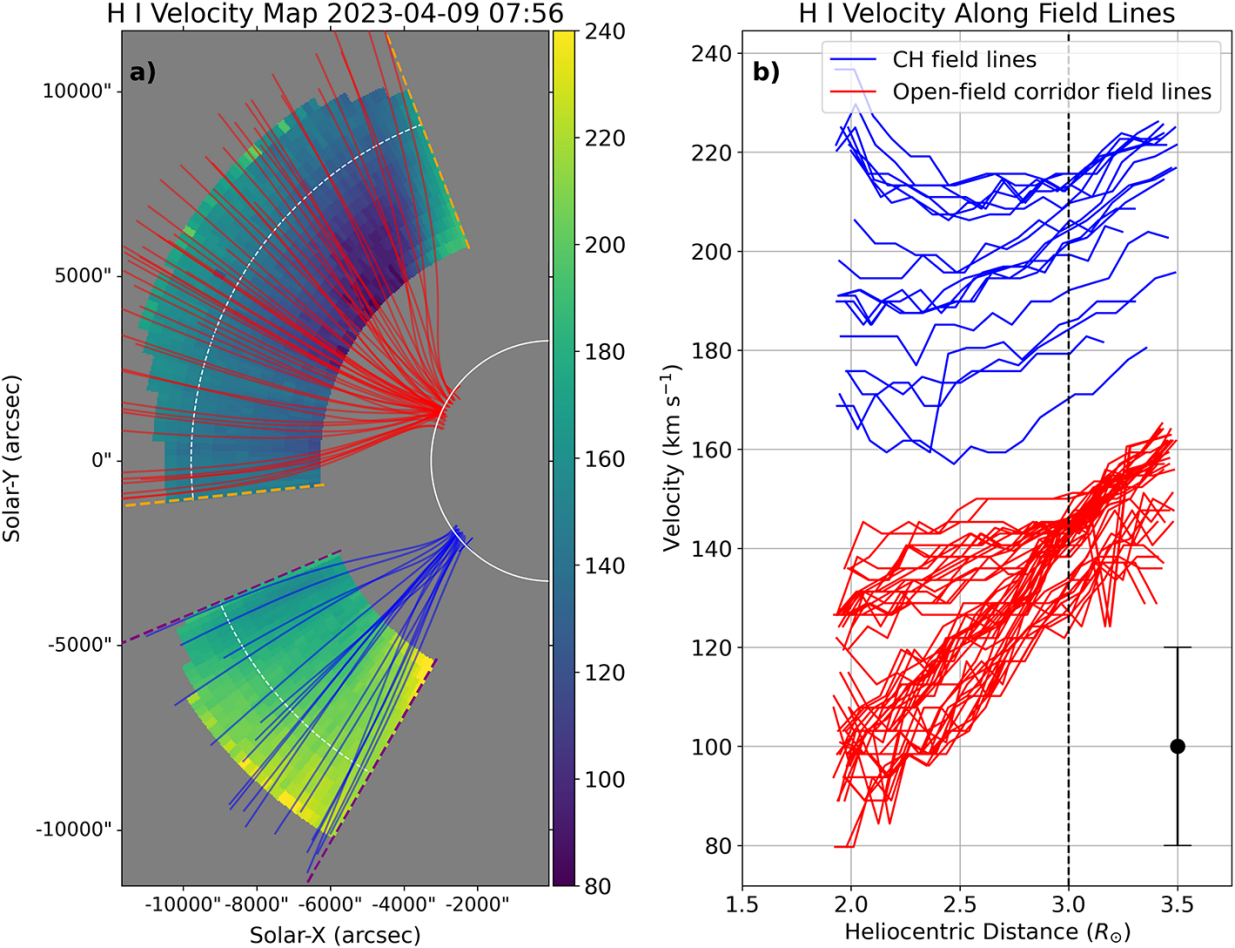
H i outflow velocity derived from the Doppler dimming method. (a) H i outflow velocity map of the solar east limb seen by Metis, with overplotted extrapolated open-field lines from the open-field corridor (red lines) and CH (blue lines). Note that our assumptions apply only to regions bounded by orange dashed lines (for the open-field corridor) and purple dashed lines (for the CH). (b) Plot of H i outflow velocity values along the open-field lines (red for the open-field corridor and blue for the CH) against the heliocentric distance. The white and black dashed lines indicate a heliocentric distance of 3 R_⊙_. The representative error of $\approx \pm 20$ km s^−1^ is indicated as a black error bar.

Figure 7 shows the results of the solar wind velocity derivation using Doppler dimming analysis. Panel a displays the H i outflow velocity map in the middle corona above the solar east limb seen by Metis, overplotted with the extrapolated field lines rooted in CH boundaries (blue) and the open-field corridor (red), similar to Figure 6. The velocity values in the regions above the open-field corridor appear to be noticeably lower than those above the CH regions. Note that a single set of assumptions is insufficient to derive the outflow velocities from broad coronal regions with different characteristics. Hence, our assumptions (e.g. T_*e*_, T_*k*_, degrees of anisotropy) only apply to regions that correspond to the open-field corridor (bounded by orange dashed lines) and the CH (bounded by purple dashed lines), and they do not apply to other regions (coloured grey in Figure 7), such as a helmet streamer near the south pole or an equatorial pseudostreamer.

The results are further highlighted in panel b where the velocity values along the open-field lines projected on the POS are plotted against the heliocentric distance. It is evident that the solar wind streams from the CH have a consistently higher speed than those from the open-field corridor at all distances. The CH solar wind has a speed in the range of $\approx 160- 240$ km s^−1^, while the open-field corridor solar wind has a speed in the range of ≈ 80 – 160 km s^−1^. The minimum speed of the CH solar wind at a low heliocentric distance ($\sim 160$ km s^−1^) is comparable to the maximum speed of the open-field corridor solar wind at the highest heliocentric distance. Both solar wind streams are moderately accelerated with increasing distance. However, the CH solar wind shows a peculiar flat or decreasing outflow velocity profile in regions below 2.5 R_⊙_. We suspect that this might be due to the assumption of uniform chromospheric Ly$\alpha $ intensity, which leads to a considerable overestimation of the outflow speed in low-intensity CH at low coronal altitudes (Dolei et al. 2019). This effect becomes less noticeable at larger heliocentric distances.

### Correlation Between Plasma and Magnetic Field Properties in the Middle Corona

**Figure 8:**
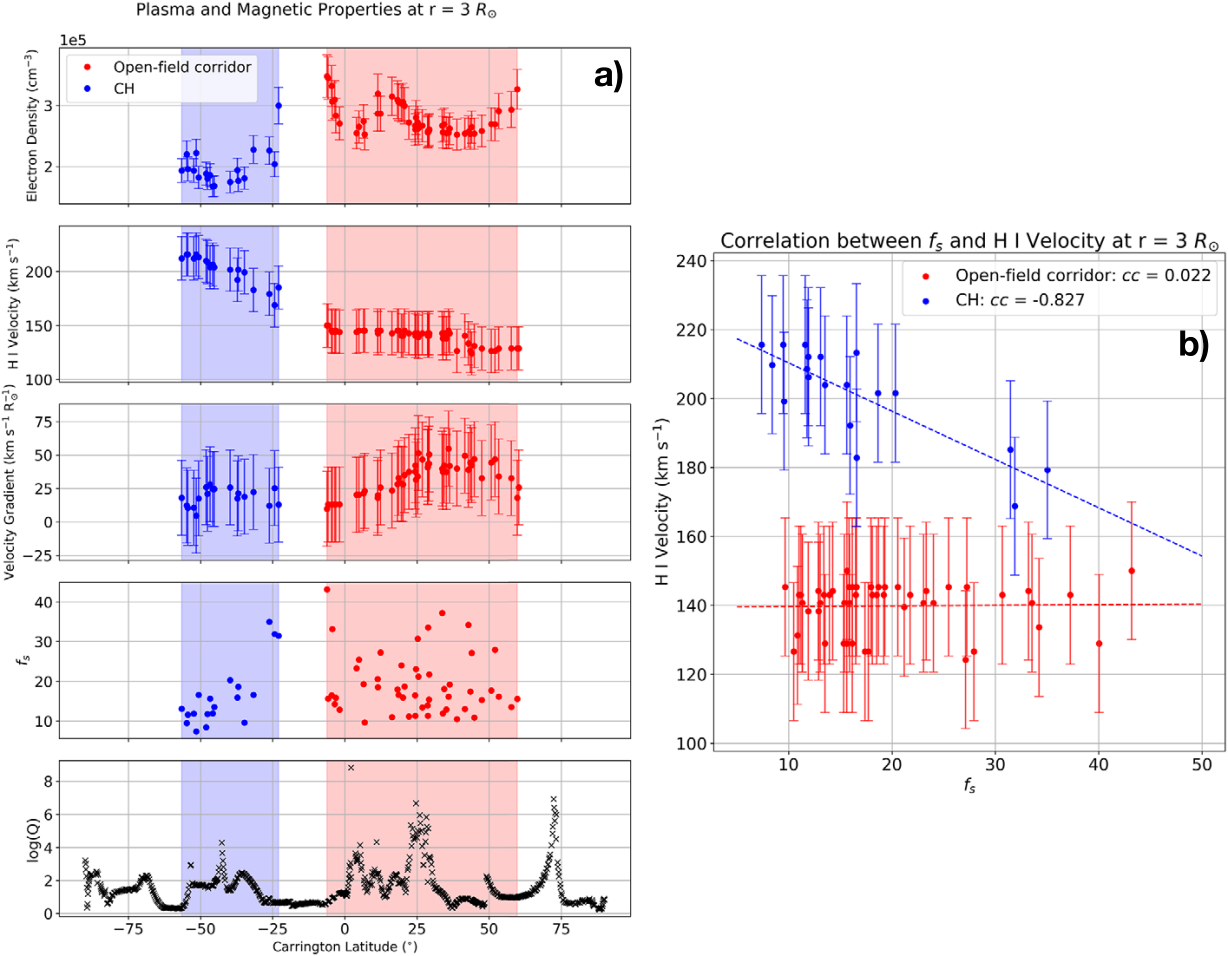
The plasma and magnetic field properties in the middle corona at a heliocentric distance of 3 R_⊙_ above the east solar limb seen by Metis. (a) Latitudinal distribution of electron density, H i outflow velocity, velocity gradient (defined in text), $f_{s}$, and logQ. The blue (red) shaded region corresponds to the latitudinal extent of the CH (open-field corridor) region. (b) Correlation between H i outflow velocity and $f_{s}$ for CH and open-field corridor solar wind. The Pearson correlation coefficient are displayed in the legend.

The electron density and outflow velocity maps, shown in Figures 6 and 7, allow us to directly compare solar wind streams arising from different coronal structures and investigate how their properties are distributed across the Carrington latitude. Figure 8 shows the plasma and magnetic properties in the middle corona above the solar east limb ($r = 3~\mathrm{R}_{\odot}$) against Carrington latitude. From panel a, we can clearly see the difference in the properties of the solar wind originating in two different source regions, with the CH solar wind (blue) having a lower electron density and higher H i outflow speed compared to the open-field corridor solar wind (red). The average electron density in the CH is $\sim 1.5$ times lower than in the open-field corridor, while the outflow speed in CH ranges from 170 to 210 km s^−1^ compared to $\approx 150$ km s^−1^ in the open-field corridor.

The average solar wind acceleration with distance is quantified as a velocity gradient, defined as $\Delta v/\Delta r$. We choose to calculate the average velocity gradient in the distance range $r=2.5-3.5~\text{R}_{\odot}$ to avoid the flat/decreasing velocity profile issue arising in the lower altitudes of the CH. The middle plot of panel a shows that the open-field corridor solar wind has a slightly higher velocity gradient than the CH solar wind, although the differences are within the estimated uncertainties.

The expansion factors $f_{s}$ of the field lines arising from the CH and the open-field corridor are generally of the same order of magnitude, with values in the range ranging ∼ 10 – 40. Meanwhile, logQ values are higher in the open-field corridor compared to the CH (see also Figure 3), especially at Carrington latitude +25^∘^ where Q values reach 10^6^.

The observed CH solar wind speeds, as inferred from the velocity of H i, seem to have a clear trend of variation with the Carrington latitude, with lower speeds near the equator and higher speeds closer to the south pole. This latitudinal variation is inverted in the distribution of $f_{s}$, with higher values near the equator and lower values closer to the pole. On the contrary, in the open-field corridor, the latitudinal distribution of the expansion factor $f_{s}$, appears to be higher and more scattered on average, without a specific latitudinal trend. This is reflected in the latitudinal distribution of the wind speed, which appears to be almost flat with small variations and values lower than in the CH region.

Panel b shows the correlation between the solar wind speed and the expansion factor $f_{s}$ in the middle corona. We find that the solar wind speed is inversely correlated with the values of $f_{s}$, with a Pearson correlation coefficient ($cc$) of -0.827. This inverse correlation is in line with the empirical relationship between the expansion factor and the solar wind speed at 1 AU (Wang and Sheeley 1990). However, we find no correlation between solar wind speed and $f_{s}$ in the open-field corridor, with a $cc$ of 0.022.

## Dynamics in the Low-Middle Corona

Fine-scale structures in the middle corona are difficult to observe in the EUV passbands, especially in the open-field regions with low density. EUV emission mainly arises from collisional processes with its intensity proportional to the density squared ($I \propto n_{e}^{2}$), which means that the EUV intensity decreases rapidly as the electron density decreases with heliocentric distance. This effect is lessened in visible-light coronagraph observation, in which the intensity depends on density ($I \propto n_{e}$, see also Eq. 2). However, the limitations of previous space-based coronagraphs leave the regions below a heliocentric distance of $\sim 2~\mathrm{R}_{\odot}$ relatively underexplored. Since the inner FOV of Metis and the outer FOV of EUI/FSI partially overlap, it is possible to seamlessly study the evolution of coronal structures from near the solar surface up to the middle corona with high spatiotemporal resolution EUV and visible-light observations during the SO’s perihelion.

**Figure 9:**
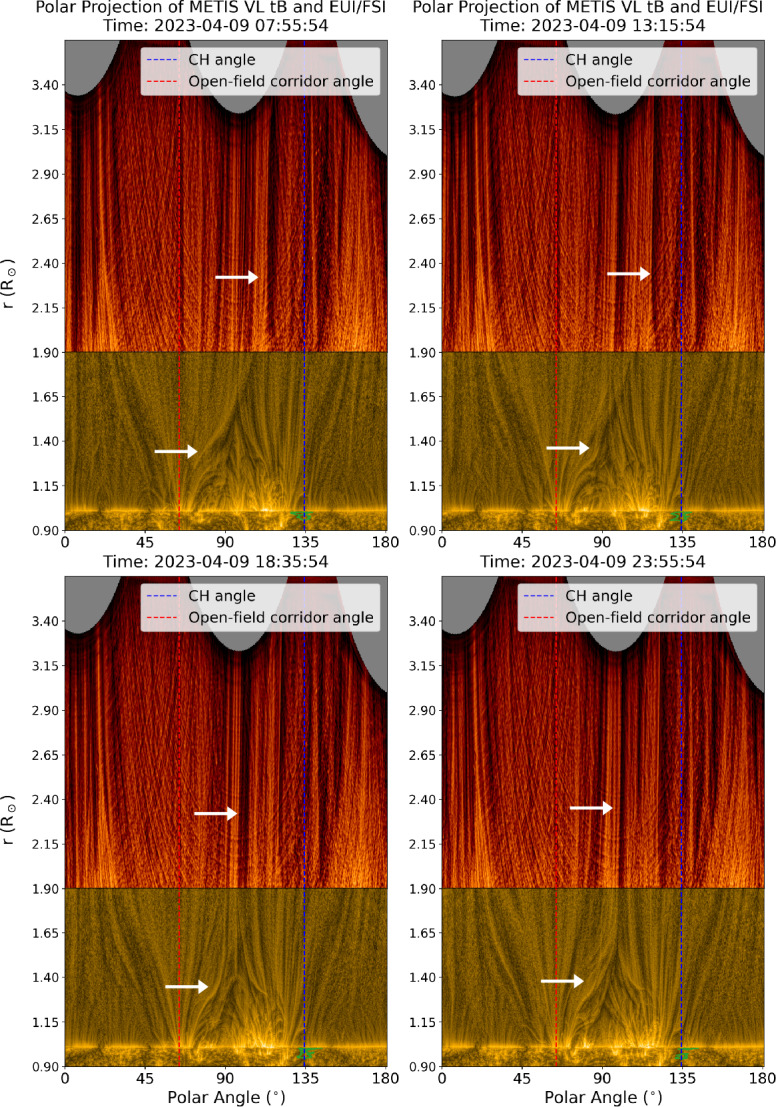
Sequence of the polar projection of combined EUI/FSI 174 Å and Metis VL tB observations of the solar east limb from a heliocentric distance of 0.90 – 3.65 R_⊙_. The red and blue dashed lines indicate the approximate polar angle of the open-field corridor and CH, respectively. The green contour marks the boundary of the CH. The white arrows are plotted to guide the eye to see the evolving boundaries of the open-field corridor in the low and middle corona. An animated version of this figure is available as Figure9Animation.mp4. The movie has a duration of 11 s and shows the evolution of the east solar limb observed from EUI/FSI and Metis from 04:56 UT to 23:56 UT.

Figure 9 shows snapshots of the east solar limb observed by EUI/FSI 174 Å and Metis VL tB at three different times during the observation date. Both observations are enhanced using the wavelet-optimised whitening method (WOW; Auchère et al. 2023) and projected into the polar coordinate system, where the polar angle starts from the solar north pole (0^∘^) and goes counterclockwise (see the yellow contour in Figure 3). The combined FOV of the composite observation covers the heliocentric distance $r = 0.90- 3.65~\mathrm{R}_{\odot}$, with SO/EUI showing the low corona in EUV and Metis showing the middle corona in visible light. The animated version of Figure 9 shows the composite SO/EUI-Metis observations of the off-limb corona above the east limb from 04:56 UT to 23:56 UT, with a cadence of 20 min.

These combined observations reveal the highly structured and extremely dynamic nature of the low and middle corona. The structures seen in the EUV and VL passbands can be smoothly connected at the height of the boundary between the two instrument FOVs ($r = 1.9~\mathrm{R}_{\odot}$). The evolution in each fine structure seen in the VL passband in the middle corona can also be traced back to the corresponding evolving EUV structures in the low corona.

There is an extended streamer near both the north and the south poles. Both streamers can be identified as the region comprised of low-emission EUV loops and brighter visible-light strands in the middle corona. The equatorial pseudostreamer (polar angle $\sim 90^{\circ}$), on the other hand, consists of numerous bright EUV loops that may result from the embedded AR (see the left panel in Figure 1) inside the pseudostreamer. The pseudostreamer cusp is located inside the EUI/FSI FOV at $r = 1.6~\mathrm{R}_{ \odot}$, which is lower than polar streamer cusps ($r$ > 2 R_⊙_). The angular width of the pseudostreamer is well-confined between the open-field corridor (red dashed line, polar angle $\sim 65^{\circ}$) and the CH (blue dashed line, polar angle $\sim 135^{\circ}$).

The open-field corridor, located to the left (north) of the pseudostreamer, is funnel-shaped and filled with numerous strands of plasma. These strands show persistent upward flows, indicating that the plasma is outflowing from the low corona to higher altitudes. The angular extent of the funnel continuously expands with heliocentric distance, tapering the shape of the north pole streamer and the equatorial pseudostreamer. For the CH on the right (south) side of the pseudostreamer, we also identify a funnel-shaped plasma outflow similar to the open-field corridor. We interpret these outflowing plasma strands as the tracers of the solar wind. However, the bright strands seen in EUV and VL may not necessarily arise from the CH as there is another open-field region at the edge of the AR located behind the limb (see panel a of Figure 2). Hence, it may be possible that those bright strands are from behind the limb and can be seen because of the LOS integration effect.

The elongated ray-like strands correspond to fine-scale structures in the open-field regions, commonly referred to as plumes (Poletto 2015) or plumelets (Uritsky et al. 2021; Morton and Cunningham 2023). These structures may extend from the low corona up to a distance of 45 R_⊙_ (e.g. DeForest, Plunkett, and Andrews 2001) and highlight the nonuniformity of plasma and magnetic field structures in the open-field region (Boe, Habbal, and Druckmüller 2020). Several twisted or helical structures, previously seen in total solar eclipse images (Druckmüller, Habbal, and Morgan 2014) can be identified near the pseudostreamer boundary in EUV observation, which may also indicate the presence of plasma instabilities.

The animated version of Figure 9 also shows the continuous reconfiguration of the structures in the low and middle corona. The reconfiguration of the structures can be seen as the formation of new loops or the deformation/brightening of existing structures in EUV or the positional shifts in visible-light strands above 1.9 R_⊙_. Since the plasma is frozen into the magnetic field in the corona, the reconfiguration of these structures can be inferred to be tracers of the evolution of magnetic field lines due to magnetic reconnection processes. The white arrows in Figure 9 point toward an example of reconfiguration of the coronal structure that is the boundary of the open-field corridor and an equatorial pseudostreamer. We observe a clear evolution of the boundary in the EUI/FSI passband over four time steps shown in Figure 9, where the shape of the boundary is significantly changed. Although the evolution is less evident in the Metis observations, we can still observe the persistent shift of the boundary (pointed by arrows) toward a lower polar angle (higher latitude). The movie version of Figure 9 reveals that these events can be ubiquitously found throughout the observation period, and they seem to be more evident at the boundaries between the plasma outflow funnel and the streamer.

## Discussion and Conclusion

The coordinated observations among the Solar Orbiter, SDO, and Hinode enable us to identify and analyse solar wind streams emanating from two different source regions: a mid-latitude coronal hole and an open-field corridor. The solar wind plasma properties are investigated in the low corona using EIS and in the middle corona using Metis. Magnetic field extrapolations based on MHD modelling are used to verify the connection between solar wind sources in the low and middle corona and to provide the global magnetic field configuration, which plays an important role in solar wind formation and acceleration.

By comparing the solar wind plasma properties (electron density and outflow velocity) from the two sources at two different solar altitudes, we find that the differences between the two solar wind streams are more pronounced in the middle corona. Plasma density and composition (as inferred by FIP bias) are very similar for both the CH and the open-field corridor solar wind, while their plasma dynamics (i.e. Doppler and nonthermal velocity) are slightly different (see Table 1). However, in the middle corona, we can clearly distinguish these two solar wind streams, with the open-field corridor solar wind generally having higher electron densities and lower outflow speeds than the CH solar wind (see Figure 8).

Note that the plasma properties in the low and middle corona are derived from different populations of plasma. This is particularly important for the derivation of the outflow velocity, which represents the dynamics of Fe ions in the low corona and of neutral hydrogen atoms in the middle corona. Although both elements can be interpreted as solar wind outflows, directly linking their dynamics is nontrivial. The feasibility of connecting the Doppler velocity from spectroscopy with the H i velocity from the Doppler dimming technique is interesting and can be explored in future work.

We acknowledge that the complex structure of the corona near solar maximum makes the interpretation of the data challenging. One of the main limitations of this work is that, although we establish the connection between EIS and Metis through a magnetic field extrapolation, it is difficult to confirm whether Metis was observing the same features as EIS. This difficulty arises from the limited FOV of EIS and also from the LOS integration effect in off-limb observations. The superimposed structures along the LOS may also considerably affect the derivation of electron density and outflow velocity. However, our results for the electron density of the CH agree with previous observations of CH at solar maximum (Withbroe 1988). Note that most of the CH electron density values reported in the previous literature (e.g. Guhathakurta et al. 1999; Hayes, Vourlidas, and Howard 2001; Morgan and Habbal 2007) are lower than our values because the previous measurements were taken in various phases of a different solar cycle (Ventura et al. 2005; Antonucci et al. 2020b). The electron density values for the open-field corridor also seem to be reasonable since they are between the values of the CHs and the equatorial streamers (see also Abbo et al. 2010). The LOS integration effect is also taken into account in the assumption for the Doppler dimming technique used to derive H i outflow velocities. Hence, our results are still valid despite these caveats.

Another limitation is that we do not have simultaneous measurements of electron and hydrogen kinetic temperature in the middle corona that complement Metis observations. This problem could be alleviated in the future by new instruments, such as the Coronal Diagnostic Experiment (CODEX; Casti et al. 2024; Gong et al. 2024), which is capable of measuring coronal temperature in the heliocentric distance range of 3 – 10 R_⊙_.

### Nature of the Open-Field Corridor

Despite the similarity in plasma composition and density, the open-field corridor and CH can still be distinguished by their differences in appearance in the EUV corona and magnetic-field properties. The magnetic flux density of the open-field corridor in the photosphere is in the order of 20 – 50 G, which is significantly higher than the CH counterpart. This disparity implies that, although both regions host an open magnetic field configuration, the magnetic environments of the open-field corridor and CHs (including surrounding boundary regions) are fundamentally different.

We find that the open-field corridor corresponds to a decayed AR, which is commonly associated with the formation of nonpolar CHs (Karachik, Pevtsov, and Abramenko 2010; Petrie and Haislmaier 2013). The corridor is not obviously darker than the surrounding region in AIA 193 Å, which could be the result of the LOS effect of several bright plumes (see panel a in Figure 4) and the remnants of AR loops that outshine the dark regions adjacent to them. The brighter EUV emission of the open-field corridor compared to the CH, even though both regions have a similar electron density (see Table 1), may also imply that it has a somewhat higher electron temperature. However, since the derived density is taken from measurements at CH boundary regions, the actual CH density may also be slightly lower than that of the open-field corridor, resulting in lower emissions. The higher electron temperature and stronger magnetic field in the open-field corridor suggest that there are additional heating mechanisms that may affect the formation of solar wind emanating from it.

Wang and Ko (2019) found that some equatorial CHs during the solar maximum may not appear dark because of nearby bright loops from a nearby AR or its remnant. They also find a magnetic field strength of the order of 30 G and a high expansion factor ($f_{s}$ > 9). The properties of the open-field corridor in our observation are comparable to this result, suggesting that it could be categorised as a thin mid-latitude CH.

However, the open-field corridor can also be compared with the dark channel near an AR observed by Baker et al. (2023). In particular, the plasma density, plasma dynamics, and magnetic-field properties inside the open-field corridor are approximately consistent with their analysis. Note that the FIP bias in the Baker et al. (2023) channel is higher than in our observation, probably due to close proximity to an AR. Baker et al. (2023) interpreted their dark channel as the narrow open-field corridor associated with the S-web model, due to its extremely high expansion factor (up to 300), topological robustness, association with a pseudostreamer, and also the very low solar wind speeds observed in situ. These characteristics are also applicable to our observations, suggesting that the open-field corridor could be associated with the S-web structure in the solar corona. The high values of logQ above that region (see Figure 3) also support this argument since they indicate the existence of separatrix surfaces and QSLs.

Therefore, we propose that this open-field corridor is a narrow mid-latitude coronal hole that forms part of the S-web structure due to its high squashing factor and the association with the leg of a pseudostreamer. Note that S-web is defined purely based on magnetic modelling, and it can encompass various structures in the low corona, including both CH and AR (Chitta et al. 2023a; Baker et al. 2023).

The open-field corridor also serves as direct evidence that not all open-field lines originate from CHs traditionally defined based on EUV observations. Asvestari et al. (2024) showed that the open-field regions derived from various coronal models (including PSI-MAS) are considerably mismatched with CH areas extracted from EUV image, even during solar minimum, where CHs are better observed. We suspect that the mismatch will become even more evident as the solar cycle progresses toward maximum, as shown from our analysis in which the open-field corridor does not appear dark in AIA 193 Å. Moreover, Boe, Habbal, and Druckmüller (2020) showed that open-field lines seem to be abundant in total solar eclipse observations even without the presence of CHs on the solar disc. This mismatch between modelling and observations needs to be further addressed in order to resolve the open flux problem (Linker et al. 2017).

### Reconnection Dynamics Driving Solar Wind Variability

By using the visible-light pB inversion and Doppler dimming technique to analyse the data from Metis, we can derive the solar wind electron density and outflow velocity map in the middle corona and can directly compare the distribution of solar wind properties emanating from the CH and the open-field corridor. We obtain two key results from the analysis. First, we find that the electron density and solar wind velocity arising from two different sources are distinctively different in the middle corona compared to the low corona, suggesting that certain processes arise between the low and middle corona that drive these differences. Second, we also find that the speed of the solar wind from the open-field corridor does not appear to be inversely correlated with the expansion factor in the middle corona ($cc = 0.022$), unlike the solar wind from the CH ($cc = -0.827$). In the expansion factor framework, the solar wind heating distribution and subsequently, the solar wind speed depend on the geometry of the flux tubes. Therefore, this noncorrelation between solar wind speed and expansion factor suggests that there should be other phenomena that lessen the effects of flux tube geometry on solar wind speed.

The structure of the low-middle corona is complex and dynamic, as suggested by the coobservation of EUI/FSI and Metis (Figure 9 and accompanying animation) and the calculation of the squashing factor logQ (Figure 3), which serves as a proxy for the complex magnetic environment preferred for reconnection. By comparing Figures 3 and 9, it is evident that the boundary between the equatorial pseudostreamer and the expanding funnel of the open-field corridor approximately corresponds to high logQ regions, while the CH region generally has a lower logQ (see also Figure 8). Therefore, we can also infer that reconnection may occur more readily inside the open-field corridor and its boundary than in the CH because of the more complex magnetic field structure.

Numerous cases of plasma structure reconfiguration observed inside or nearby open-field regions indicate that the solar wind outflow is not steady and smooth but rather is constantly undergoing the process of magnetic reconnection, especially along the boundaries of different magnetic domains (e.g. the edge of pseudostreamer) indicated by the high logQ arcs. Therefore, we hypothesise that these ubiquitous reconnection processes play an important role in driving the variability of solar wind streams.

Chitta et al. (2023a) identified highly structured elongated features in EUV observations of the middle corona, which they called the ‘coronal web’. In their observations, the coronal web seemed to emanate from a large region of complex magnetic structures with high squashing factors, and each individual coronal web continuously undergoes reconfiguration. Hence, they interpreted the coronal web as the direct imprint of S-web dynamics in the middle corona that drives the slow solar wind through magnetic reconnection. Our observation of structured plasma outflow strands in EUV and VL images could correspond to similar coronal web features reported in Chitta et al. (2023a) since both structures are similar in dynamics, spatial extent, and association with high squashing factors. In particular, in the open-field corridor, we notice that the reconfiguration events are more evident and the squashing factor values are considerably higher compared to the CH region. This might imply that there are numerous solar wind streams generated through reconnection events in the low and middle corona in our observations.

The solar wind streams generated in this way will likely have different properties compared to the solar wind originating from the sources in the low corona (i.e. upflow regions). For example, the plasma density is likely to be intermediate between the values in the two reconnecting structures, and the speed of plasma may also be related to the energetics of each reconnection event itself rather than the expansion factor of the flux tubes. These newly generated solar wind streams will inevitably mix with existing streams, and this might help explain why the difference in properties of solar wind from between the open-field corridor and the CH is more pronounced in the middle corona.

Therefore, our work highlights the importance of magnetic reconnection for solar wind formation and acceleration, in line with the S-web model. The highly dynamic nature of the middle corona and the increasingly complex magnetic field structures as the solar cycle progresses towards the maximum serve as the ideal environment for reconnection to occur more ubiquitously and to directly impact the variability of solar wind. However, the intrinsic properties of the source regions, such as magnetic field strength and fine-scale structures inside open-field regions, are still important for solar wind variability and cannot be completely ruled out. Our work is also in line with the recent framework introduced by Viall and Borovsky (2020), which challenges the historical paradigm of solar wind bimodality by suggesting that the solar wind can go through multiple pathways involving different processes, resulting in various types of solar wind parcels in contrast to the traditional fast-slow distinction. Finally, this research also demonstrates the ability to combine spectroscopic and coronagraph observations from different vantage points, which proves to be powerful in helping us better understand the origin of the solar wind.

## Supplementary Information

Below is the link to the electronic supplementary material.

(MP4 18.4 MB)

## Data Availability

Solar Orbiter data are publicly available at the Solar Orbiter Archive (https://soar.esac.esa.int/soar). Processed Hinode/EIS data are downloaded from the U.S. Naval Research Laboratory database (https://eis.nrl.navy.mil). SDO/AIA and SDO/HMI data can be accessed from the Joint Science Operations Center (http://jsoc.stanford.edu).

## Footnotes

1 DOI.

2 https://www.isas.jaxa.jp/home/solar/hinode_op/hop.php?hop=0462.

3 https://www.predsci.com/mhdweb/data_access.php.

4 EIS Software Note: https://zenodo.org/records/6339584.

5 https://lasp.colorado.edu/lisird/data/composite_lyman_alpha.

## References

[CR1] Abbo, L., Antonucci, E., Mikić, Z., Linker, J.A., Riley, P., Lionello, R.: 2010, Characterization of the slow wind in the outer corona. *Adv. Space Res.* **46**, 1400. DOI. ADS.

[CR2] Abbo, L., Ofman, L., Antiochos, S.K., Hansteen, V.H., Harra, L., Ko, Y.-K., Lapenta, G., Li, B., Riley, P., Strachan, L., et al.: 2016, Slow solar wind: observations and modeling. *Space Sci. Rev.* **201**, 55. DOI. ADS.

[CR3] Allen, L.A., Habbal, S.R., Hu, Y.Q.: 1998, Thermal coupling of protons and neutral hydrogen in the fast solar wind. *J. Geophys. Res.* **103**, 6551. DOI. ADS.

[CR4] Alzate, N., Morgan, H., Viall, N., Vourlidas, A.: 2021, Connecting the low to the high corona: a method to isolate transients in STEREO/COR1 images. *Astrophys. J.* **919**, 98. DOI. ADS.

[CR5] Antiochos, S.K., Mikić, Z., Titov, V.S., Lionello, R., Linker, J.A.: 2011, A model for the sources of the slow solar wind. *Astrophys. J.* **731**, 112. DOI. ADS.

[CR6] Antonucci, E.: 2006, Wind in the solar corona: dynamics and composition. *Space Sci. Rev.* **124**, 35. DOI. ADS.

[CR7] Antonucci, E., Abbo, L., Dodero, M.A.: 2005, Slow wind and magnetic topology in the solar minimum corona in 1996-1997. *Astron. Astrophys.* **435**, 699. DOI. ADS.

[CR8] Antonucci, E., Dodero, M.A., Giordano, S., Krishnakumar, V., Noci, G.: 2004, Spectroscopic measurement of the plasma electron density and outflow velocity in a polar coronal hole. *Astron. Astrophys.* **416**, 749. DOI. ADS.

[CR9] Antonucci, E., Harra, L., Susino, R., Telloni, D.: 2020b, Observations of the solar corona from space. *Space Sci. Rev.* **216**, 117. DOI. ADS.

[CR10] Antonucci, E., Romoli, M., Andretta, V., Fineschi, S., Heinzel, P., Moses, J.D., Naletto, G., Nicolini, G., Spadaro, D., Teriaca, L., et al.: 2020a, Metis: the solar orbiter visible light and ultraviolet coronal imager. *Astron. Astrophys.* **642**, A10. DOI. ADS.

[CR11] Antonucci, E., Downs, C., Capuano, G.E., Spadaro, D., Susino, R., Telloni, D., Andretta, V., Da Deppo, V., De Leo, Y., Fineschi, S., et al.: 2023, Slow wind belt in the quiet solar corona. *Phys. Plasmas* **30**, 022905. DOI. ADS.

[CR12] Astropy Collaboration, Robitaille, T.P., Tollerud, E.J., Greenfield, P., Droettboom, M., Bray, E., Aldcroft, T., Davis, M., Ginsburg, A., Price-Whelan, A.M., et al. 2013, Astropy: a community Python package for astronomy. *Astron. Astrophys.* **558**, A33. DOI. ADS.

[CR13] Asvestari, E., Temmer, M., Caplan, R.M., Linker, J.A., Heinemann, S.G., Pinto, R.F., Henney, C.J., Arge, C.N., Owens, M.J., Madjarska, M.S., et al.: 2024, Coronal models and detection of the open magnetic field. *Astrophys. J.* **971**, 45. DOI. ADS.

[CR14] Auchère, F.: 2005, Effect of the H I Ly chromospheric flux anisotropy on the total intensity of the resonantly scattered coronal radiation. *Astrophys. J.* **622**, 737. DOI. ADS.

[CR15] Auchère, F., Soubrié, E., Pelouze, G., Buchlin, É.: 2023, Image enhancement with wavelet-optimized whitening. *Astron. Astrophys.* **670**, A66. DOI. ADS.

[CR16] Baker, D., van Driel-Gesztelyi, L., Mandrini, C.H., Démoulin, P., Murray, M.J.: 2009, Magnetic reconnection along quasi-separatrix layers as a driver of ubiquitous active region outflows. *Astrophys. J.* **705**, 926. DOI. ADS.

[CR17] Baker, D., Démoulin, P., Yardley, S.L., Mihailescu, T., van Driel-Gesztelyi, L., D’Amicis, R., Long, D.M., To, A.S.H., Owen, C.J., Horbury, T.S., et al.: 2023, Observational evidence of S-web source of the slow solar wind. *Astrophys. J.* **950**, 65. DOI. ADS.

[CR18] Bale, S.D., Badman, S.T., Bonnell, J.W., Bowen, T.A., Burgess, D., Case, A.W., Cattell, C.A., Chandran, B.D.G., Chaston, C.C., Chen, C.H.K., et al.: 2019, Highly structured slow solar wind emerging from an equatorial coronal hole. *Nature* **576**, 237. DOI. ADS. 31802007 10.1038/s41586-019-1818-7

[CR19] Bale, S.D., Drake, J.F., McManus, M.D., Desai, M.I., Badman, S.T., Larson, D.E., Swisdak, M., Horbury, T.S., Raouafi, N.E., Phan, T., et al.: 2023, Interchange reconnection as the source of the fast solar wind within coronal holes. *Nature* **618**, 252. DOI. ADS. 37286648 10.1038/s41586-023-05955-3 PMC10247371

[CR20] Barnes, W., Cheung, M., Bobra, M., Boerner, P., Chintzoglou, G., Leonard, D., Mumford, S., Padmanabhan, N., Shih, A., Shirman, N., et al.: 2020, aiapy: a python package for analyzing solar EUV image data from AIA. *J. Open Source Softw.* **5**, 2801. DOI. ADS.

[CR21] Boe, B., Downs, C., Habbal, S.: 2023, The solar minimum eclipse of 2019 July 2. III. Inferring the coronal with a radiative differential emission measure inversion. *Astrophys. J.* **951**, 55. DOI. ADS.

[CR22] Boe, B., Habbal, S., Druckmüller, M.: 2020, Coronal magnetic field topology from total solar eclipse observations. *Astrophys. J.* **895**, 123. DOI. ADS.

[CR23] Boe, B., Habbal, S., Downs, C., Druckmüller, M.: 2021, The color and brightness of the F-corona inferred from the 2019 July 2 total solar eclipse. *Astrophys. J.* **912**, 44. DOI. ADS.

[CR24] Brooks, D.H., Ugarte-Urra, I., Warren, H.P.: 2015, Full-Sun observations for identifying the source of the slow solar wind. *Nat. Commun.* **6**, 5947. DOI. ADS. 25562705 10.1038/ncomms6947 PMC4354106

[CR25] Brooks, D.H., Warren, H.P.: 2011, Establishing a connection between active region outflows and the solar wind: abundance measurements with EIS/Hinode. *Astrophys. J. Lett.* **727**, L13. DOI. ADS.

[CR26] Brooks, D.H., Warren, H.P.: 2012, The coronal source of extreme-ultraviolet line profile asymmetries in solar active region outflows. *Astrophys. J. Lett.* **760**, L5. DOI. ADS.

[CR27] Casti, M., Newmark, J.S., Kim, Y.-H., Capobianco, G., Haudemand, H., Song, D., Park, S.-H., Bong, S.-C., Cho, K., Choi, S., et al.: 2024, Polarimetric characterization of the coronal diagnostic experiment (CODEX). In: Coyle, L.E., Matsuura, S., Perrin, M.D. (eds.) *Space Telescopes and Instrumentation 2024: Optical, Infrared, and Millimeter Wave*, *Society of Photo-Optical Instrumentation Engineers (SPIE) Conference Series* **13092**, 130927O. DOI. ADS.

[CR28] Chitta, L.P., Seaton, D.B., Downs, C., DeForest, C.E., Higginson, A.K.: 2023a, Direct observations of a complex coronal web driving highly structured slow solar wind. *Nat. Astron.* **7**, 133. DOI. ADS.

[CR29] Chitta, L.P., Zhukov, A.N., Berghmans, D., Peter, H., Parenti, S., Mandal, S., Aznar Cuadrado, R., Schühle, U., Teriaca, L., Auchère, F., et al.: 2023b, Picoflare jets power the solar wind emerging from a coronal hole on the Sun. *Science* **381**, 867. DOI. ADS. 37616348 10.1126/science.ade5801

[CR30] Cranmer, S.R.: 2009, Coronal holes. *Living Rev. Solar Phys.* **6**, 3. DOI. ADS. 10.12942/lrsp-2009-3 PMC4841186 27194961

[CR31] Cranmer, S.R., Panasyuk, A.V., Kohl, J.L.: 2008, Improved constraints on the preferential heating and acceleration of oxygen ions in the extended solar corona. *Astrophys. J.* **678**, 1480. DOI. ADS.

[CR32] Cranmer, S.R., Winebarger, A.R.: 2019, The properties of the solar corona and its connection to the solar wind. *Ann. Astron. Astrophys.* **57**, 157. DOI. ADS.

[CR33] Cranmer, S.R., Kohl, J.L., Noci, G., Antonucci, E., Tondello, G., Huber, M.C.E., Strachan, L., Panasyuk, A.V., Gardner, L.D., Romoli, M., et al.: 1999, An empirical model of a polar coronal hole at solar minimum. *Astrophys. J.* **511**, 481. DOI. ADS.

[CR34] Crooker, N.U., Gosling, J.T., Kahler, S.W.: 2002, Reducing heliospheric magnetic flux from coronal mass ejections without disconnection. *J. Geophys. Res. Space Phys.* **107**, 1028. DOI. ADS.

[CR35] Culhane, J.L., Harra, L.K., James, A.M., Al-Janabi, K., Bradley, L.J., Chaudry, R.A., Rees, K., Tandy, J.A., Thomas, P., Whillock, M.C.R., et al.: 2007, The EUV imaging spectrometer for Hinode. *Solar Phys.* **243**, 19. DOI. ADS.

[CR36] D’Amicis, R., Bruno, R.: 2015, On the origin of highly Alfvénic slow solar wind. *Astrophys. J.* **805**, 84. DOI. ADS.

[CR37] D’Amicis, R., Perrone, D., Bruno, R., Velli, M.: 2021, On Alfvénic slow wind: a journey from the Earth back to the Sun. *J. Geophys. Res. Space Phys.* **126**, e28996. DOI. ADS.

[CR38] De Leo, Y., Burtovoi, A., Teriaca, L., Romoli, M., Chioetto, P., Andretta, V., Uslenghi, M., Landini, F., Susino, R., Pancrazzi, M., et al.: 2023, In-flight radiometric calibration of the metis visible light channel using stars and comparison with STEREO-A/COR2 data. *Astron. Astrophys.* **676**, A45. DOI. ADS.

[CR39] De Leo, Y., Burtovoi, A., Teriaca, L., Romoli, M., Andretta, V., Uslenghi, M., et al.: 2024, In-flight radiometric calibration of the Metis UV H i Ly- channel and comparison with UVCS data. *Astron. Astrophys.*, in press.

[CR40] DeForest, C.E., Plunkett, S.P., Andrews, M.D.: 2001, Observation of polar plumes at high solar altitudes. *Astrophys. J.* **546**, 569. DOI. ADS.

[CR41] DeForest, C.E., Howard, R.A., Velli, M., Viall, N., Vourlidas, A.: 2018, The highly structured outer solar corona. *Astrophys. J.* **862**, 18. DOI. ADS.

[CR42] Demoulin, P., Henoux, J.C., Priest, E.R., Mandrini, C.H.: 1996, Quasi-separatrix layers in solar flares. I. *Method. Astron. Astrophys.* **308**, 643. ADS.

[CR43] Dolei, S., Susino, R., Sasso, C., Bemporad, A., Andretta, V., Spadaro, D., Ventura, R., Antonucci, E., Abbo, L., Da Deppo, V., et al.: 2018, Mapping the solar wind HI outflow velocity in the inner heliosphere by coronagraphic ultraviolet and visible-light observations. *Astron. Astrophys.* **612**, A84. DOI. ADS.

[CR44] Dolei, S., Spadaro, D., Ventura, R., Bemporad, A., Andretta, V., Sasso, C., Susino, R., Antonucci, E., Da Deppo, V., Fineschi, S., et al.: 2019, Effect of the non-uniform solar chromospheric Ly radiation on determining the coronal H I outflow velocity. *Astron. Astrophys.* **627**, A18. DOI. ADS.

[CR45] Druckmüller, M., Habbal, S.R., Morgan, H.: 2014, Discovery of a new class of coronal structures in white light eclipse images. *Astrophys. J.* **785**, 14. DOI. ADS.

[CR46] Einaudi, G., Boncinelli, P., Dahlburg, R.B., Karpen, J.T.: 1999, Formation of the slow solar wind in a coronal streamer. *J. Geophys. Res.* **104**, 521. DOI. ADS.

[CR47] Feldman, U., Schühle, U., Widing, K.G., Laming, J.M.: 1998, Coronal composition above the solar equator and the North Pole as determined from spectra acquired by the SUMER instrument on SOHO. *Astrophys. J.* **505**, 999. DOI. ADS.

[CR48] Fineschi, S., Naletto, G., Romoli, M., Da Deppo, V., Antonucci, E., Moses, D., Malvezzi, A.M., Nicolini, G., Spadaro, D., Teriaca, L., et al.: 2020, Optical design of the multi-wavelength imaging coronagraph Metis for the solar orbiter mission. *Exp. Astron.* **49**, 239. DOI. ADS.

[CR49] Fisk, L.A.: 2003, Acceleration of the solar wind as a result of the reconnection of open magnetic flux with coronal loops. *J. Geophys. Res. Space Phys.* **108**, 1157. DOI. ADS.

[CR50] Freeland, S.L., Handy, B.N.: 1998, Data analysis with the SolarSoft system. *Solar Phys.* **182**, 497. DOI. ADS.

[CR51] Gabriel, A.H.: 1971, Measurements on the Lyman alpha corona (papers presented at the proceedings of the international symposium on the 1970 solar eclipse, held in Seattle, U. S. A., 18-21 June, 1971.). *Solar Phys.* **21**, 392. DOI. ADS.

[CR52] Geiss, J., Gloeckler, G., von Steiger, R.: 1995, Origin of the solar wind from composition data. *Space Sci. Rev.* **72**, 49. DOI. ADS.

[CR53] Gibson, S.E., Fludra, A., Bagenal, F., Biesecker, D., del Zanna, G., Bromage, B.: 1999, Solar minimum streamer densities and temperatures using Whole Sun Month coordinated data sets. *J. Geophys. Res.* **104**, 9691. DOI. ADS.

[CR54] Gieseler, J., Dresing, N., Palmroos, C., Freiherr von Forstner, J.L., Price, D.J., Vainio, R., Kouloumvakos, A., Rodríguez-García, L., Trotta, D., Génot, V., et al.: 2023, Solar-MACH: an open-source tool to analyze solar magnetic connection configurations. *Front. Astron. Space Sci.* **9**, 384. DOI. ADS.

[CR55] Gong, Q., Newmark, J.S., Kim, Y.-H., Casti, M., Abbo, L., Baek, J.-H., Bong, S.-C., Budinoff, J., Capobianco, G., Cho, K., et al.: 2024, CODEX optical design and alignment. In: Coyle, L.E., Matsuura, S., Perrin, M.D. (eds.) *Space Telescopes and Instrumentation 2024: Optical, Infrared, and Millimeter Wave*, *Society of Photo-Optical Instrumentation Engineers (SPIE) Conference Series* **13092**, 130922I. DOI. ADS.

[CR56] Guhathakurta, M., Fludra, A., Gibson, S.E., Biesecker, D., Fisher, R.: 1999, Physical properties of a coronal hole from a coronal diagnostic spectrometer, Mauna Loa Coronagraph, and LASCO observations during the Whole Sun Month. *J. Geophys. Res.* **104**, 9801. DOI. ADS.

[CR57] Habbal, S.R., Druckmüller, M., Morgan, H., Ding, A., Johnson, J., Druckmüllerová, H., Daw, A., Arndt, M.B., Dietzel, M., Saken, J., et al.: 2011, Thermodynamics of the solar corona and evolution of the solar magnetic field as inferred from the total solar eclipse observations of 2010 July 11. *Astrophys. J.* **734**, 120. DOI. ADS.

[CR58] Habbal, S.R., Druckmüller, M., Alzate, N., Ding, A., Johnson, J., Starha, P., Hoderova, J., Boe, B., Constantinou, S., Arndt, M., et al.: 2021, Identifying the coronal source regions of solar wind streams from total solar eclipse observations and in situ measurements extending over a solar cycle. *Astrophys. J. Lett.* **911**, L4. DOI. ADS.

[CR59] Hahn, M., Bryans, P., Landi, E., Miralles, M.P., Savin, D.W.: 2010, Properties of a polar coronal hole during the solar minimum in 2007. *Astrophys. J.* **725**, 774. DOI. ADS.

[CR60] Hara, H., Watanabe, T., Harra, L.K., Culhane, J.L., Young, P.R., Mariska, J.T., Doschek, G.A.: 2008, Coronal plasma motions near footpoints of active region loops revealed from spectroscopic observations with Hinode EIS. *Astrophys. J. Lett.* **678**, L67. DOI. ADS.

[CR61] Harris, C.R., Millman, K.J., van der Walt, S.J., Gommers, R., Virtanen, P., Cournapeau, D., Wieser, E., Taylor, J., Berg, S., Smith, N.J., et al.: 2020, Array programming with NumPy. *Nature* **585**, 357. DOI. ADS. 32939066 10.1038/s41586-020-2649-2 PMC7759461

[CR62] Hayes, A.P., Vourlidas, A., Howard, R.A.: 2001, Deriving the electron density of the solar corona from the inversion of total brightness measurements. *Astrophys. J.* **548**, 1081. DOI. ADS.

[CR63] Heinemann, S.G., Temmer, M., Heinemann, N., Dissauer, K., Samara, E., Jerčić, V., Hofmeister, S.J., Veronig, A.M.: 2019, Statistical analysis and catalog of non-polar coronal holes covering the SDO-era using CATCH. *Solar Phys.* **294**, 144. DOI. ADS.

[CR64] Heinemann, S.G., Saqri, J., Veronig, A.M., Hofmeister, S.J., Temmer, M.: 2021, Statistical approach on differential emission measure of coronal holes using the CATCH. *Catalog. Sol. Phys.* **296**, 18. DOI. ADS.

[CR65] Higginson, A.K., Antiochos, S.K., DeVore, C.R., Wyper, P.F., Zurbuchen, T.H.: 2017, Formation of heliospheric arcs of slow solar wind. *Astrophys. J. Lett.* **840**, L10. DOI. ADS.

[CR66] Hofmeister, S.J., Veronig, A., Reiss, M.A., Temmer, M., Vennerstrom, S., Vršnak, B., Heber, B.: 2017, Characteristics of low-latitude coronal holes near the maximum of solar cycle 24. *Astrophys. J.* **835**, 268. DOI. ADS.

[CR67] Hunter, J.D.: 2007, Matplotlib: a 2D graphics environment. *Comput. Sci. Eng.* **9**, 90. DOI. ADS.

[CR68] Hyder, C.L., Lites, B.W.: 1970, H Doppler brightening and Lyman- Doppler dimming in moving H prominences. *Solar Phys.* **14**, 147. DOI. ADS.

[CR69] Karachik, N.V., Pevtsov, A.A., Abramenko, V.I.: 2010, Formation of coronal holes on the ashes of active regions. *Astrophys. J.* **714**, 1672. DOI. ADS.

[CR70] Kohl, J.L., Noci, G., Cranmer, S.R., Raymond, J.C.: 2006, Ultraviolet spectroscopy of the extended solar corona. *Astron. Astrophys. Rev.* **13**, 31. DOI. ADS.

[CR71] Kosugi, T., Matsuzaki, K., Sakao, T., Shimizu, T., Sone, Y., Tachikawa, S., Hashimoto, T., Minesugi, K., Ohnishi, A., Yamada, T., et al.: 2007, The Hinode (solar-B) mission: an overview. *Solar Phys.* **243**, 3. DOI. ADS.

[CR72] Kraaikamp, E., Gissot, S., Stegen, K., Mampaey, B., Verbeeck, F., Auchère, F., Berghmans, D.: 2023, SolO/EUI Data Release 6.0 2023-01. Published by Royal Observatory of Belgium (ROB). DOI.

[CR73] Laming, J.M.: 2015, The FIP and inverse FIP effects in solar and stellar coronae. *Living Rev. Solar Phys.* **12**, 2. DOI. ADS.

[CR74] Lamy, P., Llebaria, A., Boclet, B., Gilardy, H., Burtin, M., Floyd, O.: 2020, Coronal photopolarimetry with the LASCO-C2 coronagraph over 24 years [1996 - 2019]. *Solar Phys.* **295**, 89. DOI. ADS.

[CR75] Lemen, J.R., Title, A.M., Akin, D.J., Boerner, P.F., Chou, C., Drake, J.F., Duncan, D.W., Edwards, C.G., Friedlaender, F.M., Heyman, G.F., et al.: 2012, The atmospheric imaging assembly (AIA) on the solar dynamics observatory (SDO). *Solar Phys.* **275**, 17. DOI. ADS.

[CR76] Liewer, P.C., Vourlidas, A., Stenborg, G., Howard, R.A., Qiu, J., Penteado, P., Panasenco, O., Braga, C.R.: 2023, Structure of the plasma near the heliospheric current sheet as seen by WISPR/Parker solar probe from inside the streamer belt. *Astrophys. J.* **948**, 24. DOI. ADS.

[CR77] Linker, J.A., Caplan, R.M., Downs, C., Riley, P., Mikic, Z., Lionello, R., Henney, C.J., Arge, C.N., Liu, Y., Derosa, M.L., et al.: 2017, The open flux problem. *Astrophys. J.* **848**, 70. DOI. ADS.

[CR78] Lionello, R., Linker, J.A., Mikić, Z.: 2009, Multispectral emission of the Sun during the first Whole Sun Month: magnetohydrodynamic simulations. *Astrophys. J.* **690**, 902. DOI. ADS.

[CR79] Long, D.M., Chitta, L.P., Baker, D., Hannah, I.G., Ngampoopun, N., Berghmans, D., Zhukov, A.N., Teriaca, L.: 2023, Multistage reconnection powering a solar coronal jet. *Astrophys. J.* **944**, 19. DOI. ADS.

[CR80] Mikić, Z., Linker, J.A., Schnack, D.D., Lionello, R., Tarditi, A.: 1999, Magnetohydrodynamic modeling of the global solar corona. *Phys. Plasmas* **6**, 2217. DOI. ADS.

[CR81] Mikić, Z., Downs, C., Linker, J.A., Caplan, R.M., Mackay, D.H., Upton, L.A., Riley, P., Lionello, R., Török, T., Titov, V.S., et al.: 2018, Predicting the corona for the 21 August 2017 total solar eclipse. *Nat. Astron.* **2**, 913. DOI. ADS.

[CR82] Morgan, H., Cook, A.C.: 2020, The width, density, and outflow of solar coronal streamers. *Astrophys. J.* **893**, 57. DOI. ADS.

[CR83] Morgan, H., Druckmüller, M.: 2014, Multi-scale Gaussian normalization for solar image processing. *Solar Phys.* **289**, 2945. DOI. ADS. 10.1007/s11207-014-0523-9 PMC4938016 27445418

[CR84] Morgan, H., Habbal, S.R.: 2007, The long-term stability of the visible F corona at heights of 3-6 R_⊙. *Astron. Astrophys.* **471**, L47. DOI. ADS.

[CR85] Morgan, H., Habbal, S.R., Woo, R.: 2006, The depiction of coronal structure in white-light images. *Solar Phys.* **236**, 263. DOI. ADS.

[CR86] Morton, R.J., Cunningham, R.: 2023, The fine-scale structure of polar coronal holes. *Astrophys. J.* **954**, 90. DOI. ADS.

[CR87] Müller, D., St. Cyr, O.C., Zouganelis, I., Gilbert, H.R., Marsden, R., Nieves-Chinchilla, T., Antonucci, E., Auchère, F., Berghmans, D., Horbury, T.S., et al.: 2020, The solar orbiter mission. Science overview. *Astron. Astrophys.* **642**, A1. DOI. ADS.

[CR88] Ngampoopun, N., Long, D.M., Baker, D., Green, L.M., Yardley, S.L., James, A.W., To, A.S.H.: 2023, The merging of a coronal dimming and the southern polar coronal hole. *Astrophys. J.* **950**, 150. DOI. ADS.

[CR89] Noci, G., Kohl, J.L., Withbroe, G.L.: 1987, Solar wind diagnostics from Doppler-enhanced scattering. *Astrophys. J.* **315**, 706. DOI. ADS.

[CR90] Pesnell, W.D., Thompson, B.J., Chamberlin, P.C.: 2012, The solar dynamics observatory (SDO). *Solar Phys.* **275**, 3. DOI. ADS.

[CR91] Petrie, G.J.D., Haislmaier, K.J.: 2013, Low-latitude coronal holes, decaying active regions, and global coronal magnetic structure. *Astrophys. J.* **775**, 100. DOI. ADS.

[CR92] Poletto, G.: 2015, Solar coronal plumes. *Living Rev. Solar Phys.* **12**, 7. DOI. ADS.

[CR93] PSI: 2024 MAPFL, GitHub.

[CR94] Raouafi, N.E., Stenborg, G., Seaton, D.B., Wang, H., Wang, J., DeForest, C.E., Bale, S.D., Drake, J.F., Uritsky, V.M., Karpen, J.T., et al.: 2023, Magnetic reconnection as the driver of the solar wind. *Astrophys. J.* **945**, 28. DOI. ADS.

[CR95] Riley, P., Lionello, R., Caplan, R.M., Downs, C., Linker, J.A., Badman, S.T., Stevens, M.L.: 2021, Using Parker solar probe observations during the first four perihelia to constrain global magnetohydrodynamic models. *Astron. Astrophys.* **650**, A19. DOI. ADS.

[CR96] Rochus, P., Auchère, F., Berghmans, D., Harra, L., Schmutz, W., Schühle, U., Addison, P., Appourchaux, T., Aznar Cuadrado, R., Baker, D., et al.: 2020, The solar orbiter EUI instrument: the extreme ultraviolet imager. *Astron. Astrophys.* **642**, A8. DOI. ADS.

[CR97] Romoli, M., Antonucci, E., Andretta, V., Capuano, G.E., Da Deppo, V., De Leo, Y., Downs, C., Fineschi, S., Heinzel, P., Landini, F., et al.: 2021, First light observations of the solar wind in the outer corona with the Metis coronagraph. *Astron. Astrophys.* **656**, A32. DOI. ADS.

[CR98] Russano, G., Andretta, V., De Leo, Y., Teriaca, L., Uslenghi, M., Giordano, S., Telloni, D., Heinzel, P., Jejčič, S., Abbo, L., et al.: 2024, Eruptive events with exceptionally bright emission in H I Ly- observed by the Metis coronagraph. *Astron. Astrophys.* **683**, A191. DOI. ADS.

[CR99] Sakao, T., Kano, R., Narukage, N., Kotoku, J., Bando, T., DeLuca, E.E., Lundquist, L.L., Tsuneta, S., Harra, L.K., Katsukawa, Y., et al.: 2007, Continuous plasma outflows from the edge of a solar active region as a possible source of solar wind. *Science* **318**, 1585. DOI. ADS. 18063788 10.1126/science.1147292

[CR100] Scherrer, P.H., Schou, J., Bush, R.I., Kosovichev, A.G., Bogart, R.S., Hoeksema, J.T., Liu, Y., Duvall, T.L., Zhao, J., Title, A.M., et al.: 2012, The helioseismic and magnetic imager (HMI) investigation for the solar dynamics observatory (SDO). *Solar Phys.* **275**, 207. DOI. ADS. 10.1007/s11207-018-1259-8 PMC6445534 31007294

[CR101] Scott, R.B., Pontin, D.I., Wyper, P.F.: 2019, Magnetic structures at the boundary of the closed corona: a semi-automated study of S-web morphology. *Astrophys. J.* **882**, 125. DOI. ADS.

[CR102] Seaton, D.B., Hughes, J.M., Tadikonda, S.K., Caspi, A., DeForest, C.E., Krimchansky, A., Hurlburt, N.E., Seguin, R., Slater, G.: 2021, The Sun’s dynamic extended corona observed in extreme ultraviolet. *Nat. Astron.* **5**, 1029. DOI. ADS.

[CR103] Sheeley, J.N.R., Lee, D.D.-H., Casto, K.P., Wang, Y.-M., Rich, N.B.: 2009, The structure of streamer blobs. *Astrophys. J.* **694**, 1471. DOI. ADS.

[CR104] Spadaro, D., Susino, R., Ventura, R., Vourlidas, A., Landi, E.: 2007, Physical parameters of a mid-latitude streamer during the declining phase of the solar cycle. *Astron. Astrophys.* **475**, 707. DOI. ADS.

[CR105] Stansby, D., Matteini, L., Horbury, T.S., Perrone, D., D’Amicis, R., Berčič, L.: 2020, The origin of slow Alfvénic solar wind at solar minimum. *Mon. Not. Roy. Astron. Soc.* **492**, 39. DOI. ADS.

[CR106] SunPy Community, Barnes, W.T., Bobra, M.G., Christe, S.D., Freij, N., Hayes, L.A., Ireland, J., Mumford, S., Perez-Suarez, D., Ryan, D.F., et al.: 2020, The SunPy project: open source development and status of the version 1.0 core package. *Astrophys. J.* **890**, 68. DOI. ADS.

[CR107] Susino, R., Ventura, R., Spadaro, D., Vourlidas, A., Landi, E.: 2008, Physical parameters along the boundaries of a mid-latitude streamer and in its adjacent regions. *Astron. Astrophys.* **488**, 303. DOI. ADS.

[CR108] Tian, H., McIntosh, S.W., De Pontieu, B., Martínez-Sykora, J., Sechler, M., Wang, X.: 2011, Two components of the solar coronal emission revealed by extreme-ultraviolet spectroscopic observations. *Astrophys. J.* **738**, 18. DOI. ADS.

[CR109] Tian, H., Harra, L., Baker, D., Brooks, D.H., Xia, L.: 2021, Upflows in the upper solar atmosphere. *Solar Phys.* **296**, 47. DOI. ADS.

[CR110] Titov, V.S.: 2007, Generalized squashing factors for covariant description of magnetic connectivity in the solar corona. *Astrophys. J.* **660**, 863. DOI. ADS.

[CR111] Titov, V.S., Mikić, Z., Linker, J.A., Lionello, R., Antiochos, S.K.: 2011, Magnetic topology of coronal hole linkages. *Astrophys. J.* **731**, 111. DOI. ADS.

[CR112] Uritsky, V.M., DeForest, C.E., Karpen, J.T., DeVore, C.R., Kumar, P., Raouafi, N.E., Wyper, P.F.: 2021, Plumelets: dynamic filamentary structures in solar coronal plumes. *Astrophys. J.* **907**, 1. DOI. ADS.

[CR113] Uslenghi, M., Andretta, V., Teriaca, L., Heerlein, K., Nicolini, G., Pancrazzi, M., Romoli, M., Farina, S., Abbo, L., Burtovoi, A., Casini, C., De Leo, Y., Frassati, F., Jerse, G., Landini, F., Russano, G., Sasso, C., Susino, R.: 2024, Characterization of the dark signal of the solar orbiter/metis detectors. In: Holland, A.D., Minoglou, K. (eds.) *X-Ray, Optical, and Infrared Detectors for Astronomy XI*, *Society of Photo-Optical Instrumentation Engineers (SPIE) Conference Series* **13103**, 1310324. DOI. ADS.

[CR114] van de Hulst, H.C.: 1950, The electron density of the solar corona. *Bull. Astron. Inst. Neth.* **11**, 135. ADS.

[CR115] van Driel-Gesztelyi, L., Culhane, J.L., Baker, D., Démoulin, P., Mandrini, C.H., DeRosa, M.L., Rouillard, A.P., Opitz, A., Stenborg, G., Vourlidas, A., et al.: 2012, Magnetic topology of active regions and coronal holes: implications for coronal outflows and the solar wind. *Solar Phys.* **281**, 237. DOI. ADS.

[CR116] Vásquez, A.M., van Ballegooijen, A.A., Raymond, J.C.: 2003, The effect of proton temperature anisotropy on the solar minimum corona and wind. *Astrophys. J.* **598**, 1361. DOI. ADS.

[CR117] Ventura, R., Spadaro, D., Cimino, G., Romoli, M.: 2005, Streamers and adjacent regions observed by UVCS/SOHO: a comparison between different phases of solar activity. *Astron. Astrophys.* **430**, 701. DOI. ADS.

[CR118] Ventura, R., Antonucci, E., Downs, C., Romano, P., Susino, R., Spadaro, D., Telloni, D., Guglielmino, S.L., Capuano, G., Andretta, V., et al.: 2023, Recurrent solar density transients in the slow wind observed with the Metis coronagraph. *Astron. Astrophys.* **675**, A170. DOI. ADS.

[CR119] Viall, N.M., Borovsky, J.E.: 2020, Nine outstanding questions of solar wind physics. *J. Geophys. Res. Space Phys.* **125**, e26005. DOI. ADS. 10.1029/2018JA026005 PMC7380306 32728511

[CR120] Virtanen, P., Gommers, R., Oliphant, T.E., Haberland, M., Reddy, T., Cournapeau, D., Burovski, E., Peterson, P., Weckesser, W., Bright, J., et al.: 2020, SciPy 1.0: fundamental algorithms for scientific computing in Python. *Nat. Methods* **17**, 261. DOI. ADS. 32015543 10.1038/s41592-019-0686-2 PMC7056644

[CR121] Wang, Y.-M., Ko, Y.-K.: 2019, Observations of slow solar wind from equatorial coronal holes. *Astrophys. J.* **880**, 146. DOI. ADS.

[CR122] Wang, Y.-M., Sheeley, J.N.R.: 1990, Solar wind speed and coronal flux-tube expansion. *Astrophys. J.* **355**, 726. DOI. ADS.

[CR123] Wang, Y.-M., Sheeley, J.N.R., Rich, N.B.: 2007, Coronal pseudostreamers. *Astrophys. J.* **658**, 1340. DOI. ADS.

[CR124] Weberg, M., Warren, H., Crump, N., Barnes, W.: 2023, EISPAC - the EIS Python analysis code. *J. Open Source Softw.* **8**, 4914. DOI. ADS.

[CR125] West, M.J., Seaton, D.B., Wexler, D.B., Raymond, J.C., Del Zanna, G., Rivera, Y.J., Kobelski, A.R., Chen, B., DeForest, C., Golub, L., et al.: 2023, Defining the middle corona. *Solar Phys.* **298**, 78. DOI. ADS. 10.1007/s11207-023-02170-1 PMC10267282 37325237

[CR126] Withbroe, G.L.: 1988, The temperature structure, mass, and energy flow in the corona and inner solar wind. *Astrophys. J.* **325**, 442. DOI. ADS.

[CR127] Withbroe, G.L., Kohl, J.L., Weiser, H., Munro, R.H.: 1982, Probing the solar wind acceleration region using spectroscopic techniques. *Space Sci. Rev.* **33**, 17. DOI. ADS.

[CR128] Yardley, S.L., Brooks, D.H., Baker, D.: 2021, Widespread occurrence of high-velocity upflows in solar active regions. *Astron. Astrophys.* **650**, L10. DOI. ADS.

[CR129] Yardley, S.L., Brooks, D.H., D’Amicis, R., Owen, C.J., Long, D.M., Baker, D., Démoulin, P., Owens, M.J., Lockwood, M., Mihailescu, T., et al.: 2024, Multi-source connectivity as the driver of solar wind variability in the heliosphere. *Nat. Astron.* DOI. ADS. 10.1038/s41550-024-02278-9 PMC11335567 39175533

[CR130] Young, P.R., Muglach, K.: 2014, Solar dynamics observatory and Hinode observations of a blowout jet in a coronal hole. *Solar Phys.* **289**, 3313. DOI. ADS.

